# Glycosylation-dependent galectin-1/neuropilin-1 interactions promote liver fibrosis through activation of TGF-β- and PDGF-like signals in hepatic stellate cells

**DOI:** 10.1038/s41598-017-11212-1

**Published:** 2017-09-08

**Authors:** Ming-Heng Wu, Yuh-Ling Chen, Kuen-Haur Lee, Che-Chang Chang, Tsai-Mu Cheng, Szu-Yuan Wu, Chao-Chiang Tu, Wan-Lin Tsui

**Affiliations:** 1 Graduate Institute of Translational Medicine, College of Medical Sciences and Technology, Taipei Medical University, Taipei, Taiwan; 2 Institute of Oral Medicine, College of Medicine, National Cheng Kung University, Tainan, Taiwan; 3 Graduate Institute of Cancer Biology and Drug Discovery, College of Medical Sciences and Technology, Taipei Medical University, Taipei, Taiwan; 4 Department of Radiation Oncology, Wan Fang Hospital, Taipei Medical University, Taipei, Taiwan; 5 Department of Internal Medicine, School of Medicine, College of Medicine, Taipei Medical University, Taipei, Taiwan; 6 Department of General Surgery, Taipei Medical University Hospital, Taipei Medical University, Taipei, Taiwan; 7 Center for Cell Therapy and Regeneration Medicine, Taipei Medical University, Taipei, Taiwan

## Abstract

Concomitant expressions of glycan-binding proteins and their bound glycans regulate many pathophysiologic processes, but this issue has not been addressed in liver fibrosis. Activation of hepatic stellate cells (HSCs) is a rate-limiting step in liver fibrosis and is an important target for liver fibrosis therapy. We previously reported that galectin (Gal)-1, a β-galactoside-binding protein, regulates myofibroblast homeostasis in oral carcinoma and wound healing, but the role of Gal-1 in HSC migration and activation is unclear. Herein, we report that Gal-1 and its bound glycans were highly expressed in fibrotic livers and activated HSCs. The cell-surface glycome of activated HSCs facilitated Gal-1 binding, which upon recognition of the N-glycans on neuropilin (NRP)-1, activated platelet-derived growth factor (PDGF)- and transforming growth factor (TGF)-β-like signals to promote HSC migration and activation. In addition, blocking endogenous Gal-1 expression suppressed PDGF- and TGF-β1-induced signaling, migration, and gene expression in HSCs. Methionine and choline-deficient diet (MCD)-induced collagen deposition and HSC activation were attenuated in Gal-1-null mice compared to wild-type mice. In summary, we concluded that glycosylation-dependent Gal-1/NRP-1 interactions activate TGF-β and PDGF-like signaling to promote the migration and activation of HSCs. Therefore, targeting Gal-1/NRP-1 interactions could be developed into liver fibrosis therapy.

## Introduction

Liver fibrosis is an abnormal wound-healing response to liver injury, characterized by the excessive accumulation of extracellular matrix (ECM) proteins in the liver. Although the etiology of liver fibrosis is diverse, the convergent pathway is hepatic stellate cell (HSC) activation, a process of quiescent stellate cells trans-differentiating into activated myofibroblasts. Activated HSCs proliferate and migrate to injured sites, secreting large amounts of ECM which alter the normal architecture of the liver and initiate several positive feedback pathways that lead to liver fibrosis^1, 2^. Perpetuation of HSC activation is induced by autocrine and paracrine mediators such as platelet-derived growth factor (PDGF) and transforming growth factor (TGF)-β, which stimulate signal transduction and gene expression in activated HSCs^3, 4^. Therefore, strategies to eliminate or normalize activated HSCs are critical for liver fibrosis therapy.

Aberrant expressions of glycosyltransferase or glycosidases result in the remodeling of cell-surface glycans which generates favorable glycoconjugates for lectin (a carbohydrate-binding protein) binding. Concomitant changes in cell-surface glycans and lectin expressions regulate pathophysiologic processes and disease progression^5, 6^. Galectin-1, a β-galactoside-binding lectin, can from a dimer under certain circumstances^7^ and the carbohydrate-recognition domain (CRD) of each monomer recognizes a wide range of glycosylated receptors and regulates cellular signaling and physiologic activities^8^. For example, reduced ST6Gal1 (α2,6 sialyltransferase 1) in the vasculature of anti-vascular endothelial growth factor (VEGF)-refractory tumors facilitate Gal-1 binding to VEGF receptor 2 (VEGFR2) and preserve angiogenesis for tumor growth^9^. Different glycan-modifications of type 1 T helper (Th1), Th2, and interleukin (IL)-17-producing T cells (Th-17) regulate their susceptibility to Gal-1-induced cell death^10^.

Previous studies demonstrated that Gal-1 regulates myofibroblast activation in cancers^11, 12^, wound healing^13^, and pancreatitis^14^ suggesting Gal-1 may regulate HSC homeostasis. Gal-1 expression was elevated in fibrotic livers of hepatitis C virus (HCV) transgenic mice^15^ and in activated rat HSCs^16^. However, whether the remodeling of cell-surface glycans cooperates with Gal-1 to regulate HSC migration and activation is poorly understood. We previously reported that neuropilin (NRP)-1 is a critical receptor for Gal-1 to induce angiogenesis, vascular permeability, and wound-healing^13, 17, 18^, but the role of NRP-1 glycosylation in Gal-1 binding is not fully understood in HSCs. Therefore, this study investigated whether the glycome of activated HSCs facilitates Gal-1 binding to NRP-1 to induce HSC activation and migration, and liver fibrosis.

## Results

### Galectin-1 and its bound glycans are concordantly highly expressed in fibrotic livers and activated HSCs

**Figure 1:**
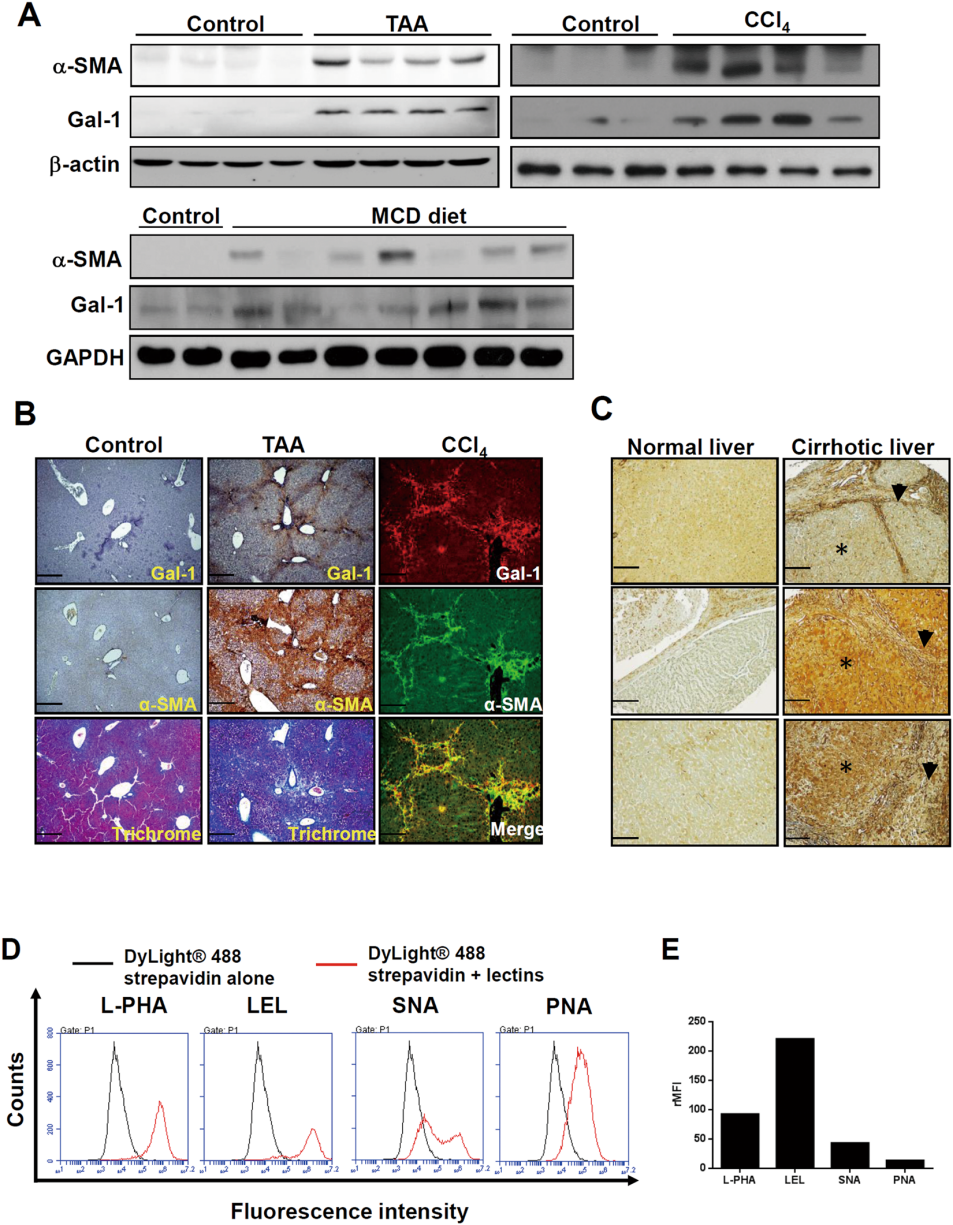
The expression of galectin-1 and its binding lectins are associated with hepatic stellate cells (HSC) activation. (**A**) Gal-1 expression is upregulated in fibrotic livers. Mouse liver fibrosis was induced by an injection of thioacetamide and carbon chloride or by feeding a methionine- and choline-deficient (MCD) diet as described in “Materials and methods”. Liver tissues were homogenized for Western blotting. (**B**) Gal-1 is expressed in areas around the portal vein and areas of bridging fibrosis in fibrotic livers of mice. Tissue slides were stained with anti-Gal-1 and anti-α-SMA antibodies and Masson’s trichrome stain. The blue color indicates collagen. The brown color indicates a region positive for Gal-1 and α-smooth muscle actin (α-SMA). For immunofluorescence analysis, the slides were incubated with anti-Gal-1 and anti-α-SMA antibodies, and the signal was visualized by Alexa Fluor-488 and -594 secondary antibodies. (**C**) Gal-1 is overexpressed in cirrhotic liver tissues compared to normal liver tissues. Normal liver and cirrhotic tissues were obtained from US Biomax (LV805). P’t represents patient. Three representative samples of normal and cirrhotic livers are respectively shown in the left and right panel. Asterisks indicate parenchymal cells, and arrowheads indicate non-parenchymal cells. (**D**) Glycosylation signatures of LX-2 cells, an activated hepatic stellate cell line. Cell surface glycans of LX-2 cells were detected by different types of lectins as described in “Materials and methods”. (**E**) Quantitation of the relative mean fluorescence intensity (rMFI) of different lectins. The rMFI was calculated by comparing the mean fluorescence intensity of different lectins to that of DyLight® 488 streptavidin alone, and results are shown as folds of change.

We first examined whether Gal-1 expression is associated with liver fibrosis and HSC activation using experimental models of liver fibrosis. Gal-1 expression was upregulated in fibrotic livers which were induced by thioacetamide (TAA), carbon tetrachloride (CCl_4_), and a methionine- and choline-deficient (MCD) diet (Fig. 1A). The serum Gal-1 concentrations of fibrotic livers were not significantly changed (Supplementary Fig. S1). IHC and immunofluorescence staining revealed that strong Gal-1 staining was spatially associated with dense collagen deposition and α-smooth muscle actin (α-SMA) expression in areas around the portal vein and areas with bridging fibrosis, suggesting that Gal-1 may regulate HSC activation (Fig. 1B). Gal-1 was also highly expressed in livers of patients with cirrhosis (Fig. 1C). Notably, two patterns of Gal-1 staining were observed: (1) Gal-1 is up-regulated in non-parenchymal regions (p’t 1). (2) Gal-1 is up-regulated in both non-parenchymal and parenchymal regions (p’t 2, 3). Immunofluorescence staining showed that Gal-1 expression correlated and co-localized with α-SMA in both patterns (Supplemental Fig. 2), indicating Gal-1 is not only highly expressed in activated HSCs but also hepatocytes. Therefore, it is believed that Gal-1 is commonly up-regulated in activated HSCs but the overexpression of Gal-1 in hepatocytes may reflect the results of long-term exposure of liver damages and the complexity of etiologies. If the prolonged liver damages continue for years, mouse livers may show a similar pattern. To understand whether cell-surface glycans in activated HSCs favor Gal-1 binding, we examined the”glycosylation signature” of LX-2 cells (immortalized and activated HSCs) using a panel of lectins that recognize specific glycan structures. N-Acetyllactosamine (lacNAc) is the minimal structure recognized by Gal-1 and can be presented as multiple units (poly-lacNAc) on N- and O-linked glycans on the cell surface through the coordinated actions of glycosyltransferases. Notably, N-acetylglucosaminyltransferase 5 (MGAT5) and core-2 β1-6-N-acetylglucosaminyltransferase 1 (GCNT1) are critical enzymes that generate the β1-6-N-acetylglucosamine (β1-6GlcNAc) as an intermediate for poly-LacNAc synthesis and extension on N- and O-glycans^19^. Intriguingly, the L-phytohemagglutinin (L-PHA)-reactive MGAT5 modified N-glycans and *Lycopersicon esculentum* lectin (LEL)-reactive poly-LacNAc were highly presented in LX-2 cells (Fig. 1D). Meanwhile, because galectin-1 binding is masked by the terminal sialic acid modification of poly-LacNac, we examined the amount of α2-6-linked sialic acid using *Sambucus nigra* agglutinin (SNA). The SNA-binding affinity was significantly lower than those of L-PHA and LEL in activated HSCs (Fig. 1D,E). Peanut agglutinin (PNA) recognizes core-1-galactosyl (β-1,3) N-acetylgalactosamine of O-glycan, which had lower affinity with LX-2 cells compared to L-PHA and LEL (Fig. 1D,E). In addition, the fibrotic livers also showed higher L-PHA binding compared to normal livers and the L-PHA binding was co-localized with α-smooth muscle actin (Supplemental Fig. S3), indicating the activated HSCs had high amount Gal-1 binding glycans *in vivo*. Interestingly, we also observed that carcinoma-associated fibroblasts of oral cancer (CAFs, a type of myofibroblast) showed higher L-PHA and LEL binding affinity compared with normal fibroblasts (Supplementary Fig. S4A). Accordingly, Gal-1’s binding affinity was higher in CAFs compared to normal fibroblasts (Supplementary Fig. S4B). Taken together, the results suggest that the glycome of activated HSCs favors Gal-1 binding which might facilitate HSC migration and activation.

**Figure 2:**
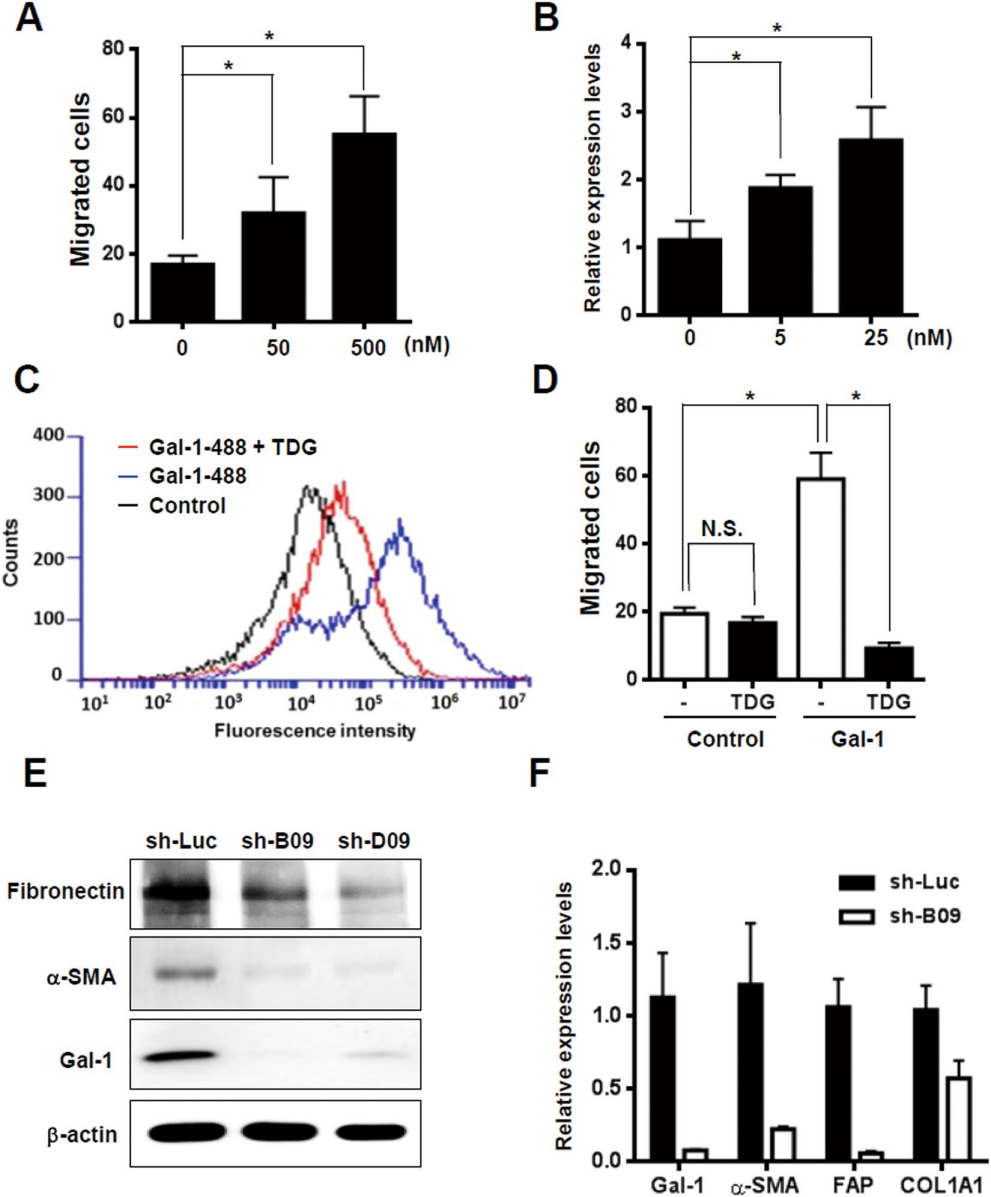
The glycome of activated hepatic stellate cells (HSCs) facilitated Gal-1 binding which induces HSC migration and activation. (**A**) Gal-1 induces the migration of LX-2 cells in a dose-dependent manner. The cell migratory ability was measured using a Boyden chamber assay. (**B**) Gal-1 induces HSC activation. LX-2 cells were starved for 24 h and then treated with different doses of the recombinant Gal-1 protein for 24 h. α-smooth muscle actin (α-SMA) expression was detected using an RT-qPCR. Relative expression levels were calculated by comparing the ΔCT values of Gal-1-treated cells to those of cells without treatment, and results are shown as folds of change. (**C**) Thiodigalactoside (TDG) inhibits Gal-1 binding with LX-2 cells. TDG (200 µM) was pre-incubated with Gal-1-488 (500 nM) for 30 min, and then LX-2 cells were incubated with the mixture for 30 min. The binding of LX-2 and Gal-1-488 was analyzed using flow cytometry. (**D**) TDG inhibits Gal-1-induced HSC migration. TDG (200 µM) was pre-incubated with Gal-1 (500 nM) for 30 min, and then LX-2 cells were treated with the mixture for 16 h. The cell migratory ability was measured using a Boyden chamber assay. (**E**,**F**) Knockdown of Gal-1 normalizes activated HSCs. LX-2 cells were infected with a lentivirus carrying Gal-1 and luciferase shRNAs (sh-B09, D09, and sh-Luc). Western blotting and an RT-qPCR were used to analyze Gal-1, α-SMA, fibroblast activation protein (FAP), and α-1 type I collagen (COL1A1) expression. Relative expression levels of individual genes were calculated by comparing the ΔCT values of sh-B09 cells to those of sh-Luc cells, and results are shown as folds of change. All of the experiments were performed in duplicate. Results are shown the mean ± SEM of three independent assays. *p < 0.05.

### Gal-1 regulates HSC migration and activation through its CRD

Because there was concomitant expression of Gal-1 and its bound glycans in activated HSCs, we examined whether Gal-1 modulates HSC migration in a glycosylation-dependent manner. LX-2 cells were used to examine the biological effect of Gal-1 on HSCs. Extracellular Gal-1 stimulation induced HSC migration and activation in a dose-dependent manner (Fig. 2A,B) and the effect was blocked by thiodigalactoside (TDG), a lactose analog, (Fig. 2C,D) which demonstrated that Gal-1-induced HSC migration was CRD-dependent. We also compared the Gal-1 induced cell migration in fibroblasts from oral carcinoma (CAFs) and their normal counterparts (NFs), because we observed that CAFs showed higher Gal-1 binding affinity compared with NFs (Supplemental Fig. S4A,B). Gal-1 induced higher migration ability in CAFs than NFs (Supplemental Fig. S4C), which indicates that the glycome of activated fibroblasts favor Gal-1 binding and Gal-1 induced migration. The Gal-1’s effect on the perpetuation of HSC activation was examined by silencing endogenous Gal-1 in LX-2 cells, because they express high levels of Gal-1 and myofibroblast markers. Blocking Gal-1 expression reversed the characteristics of activated HSCs as evidenced by reduced α-SMA, collagen, FAP, and fibronectin (Fig. 2E,F). Therefore, the results demonstrate that Gal-1 is a critical regulator of HSC migration through its CRD and is required for the perpetuation of HSC activation.

### Both N- and O-glycosylation is required for Gal-1-induced HSC migration

**Figure 3:**
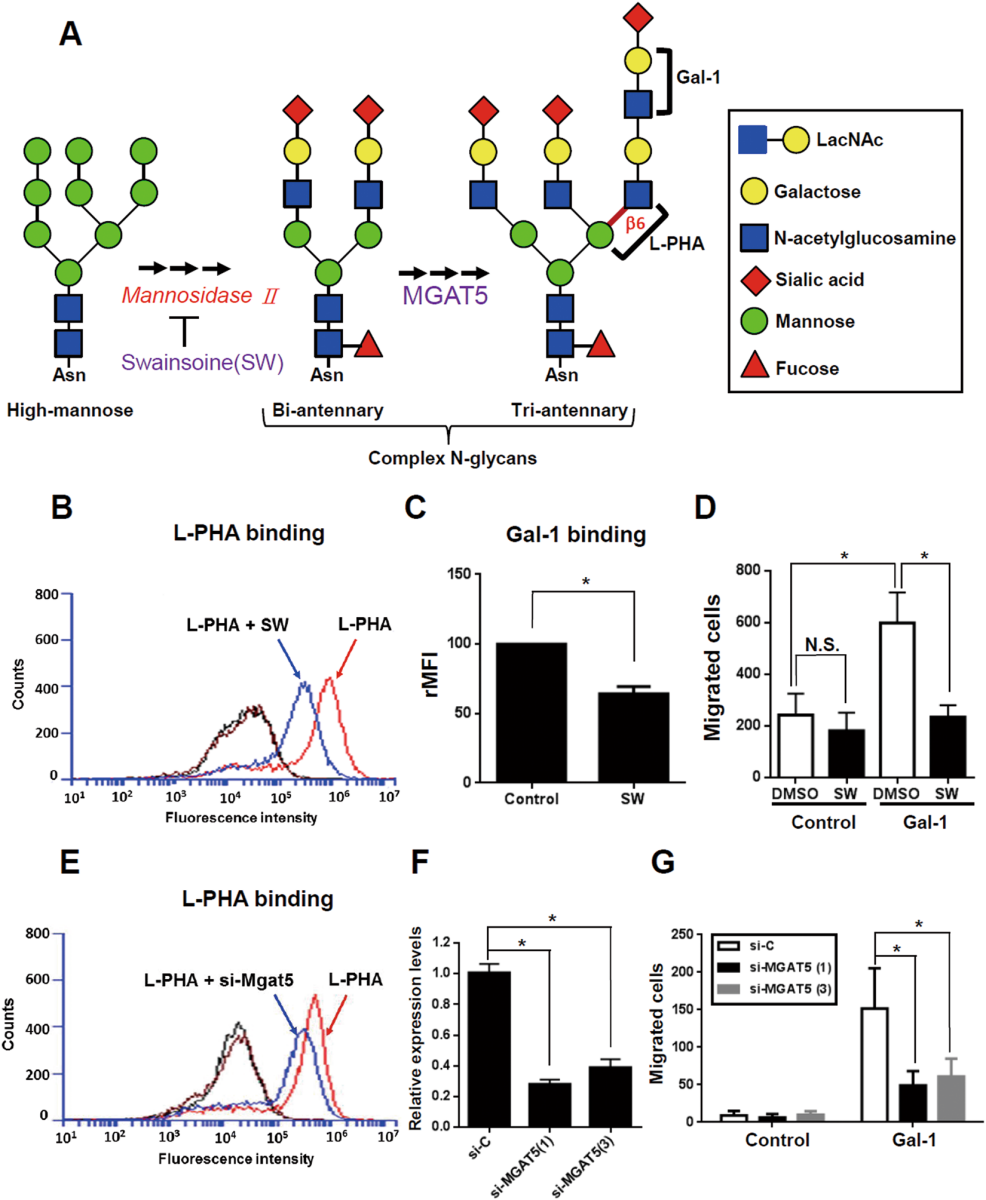
Blocking MGAT5-mediated N-glycosylation suppresses Gal-1-induced hepatic stellate cell (HSC) migration. (**A**) Schematic representation of N-glycan biosynthesis. (**B**) An N-glycan inhibitor (swainsonine, SW) inhibits L-PHA binding to LX-2 cells. (**C**) SW inhibits Gal-1 binding to LX-2 cells. (**D**) SW suppresses Gal-1-induced LX-2 cell migration. (**E**) Knockdown of MGAT5 inhibits L-PHA binding to LX-2 cells. (**F**) MGAT5 siRNAs suppress MGAT5 expression. (**G**) MGAT5 siRNAs inhibit Gal-1-induced LX-2 cell migration. LX-2 cells were treated with SW or transfected with MGAT5 siRNA for 48 h followed by incubation with L-PHA and Gal-1-488 to detect their binding to LX-2 cells using flow cytometry. The black line represents the binding of LX-2 cells with DyLight® 488 strepavidin alone and the brown line represents LX-2 cells without staining. To measure the migratory ability, cells were treated with 500 nM Gal-1 for 16 h. Migrated cells were counted, and results are presented as the mean ± SEM of three independent experiments. *p < 0.05.

**Figure 4:**
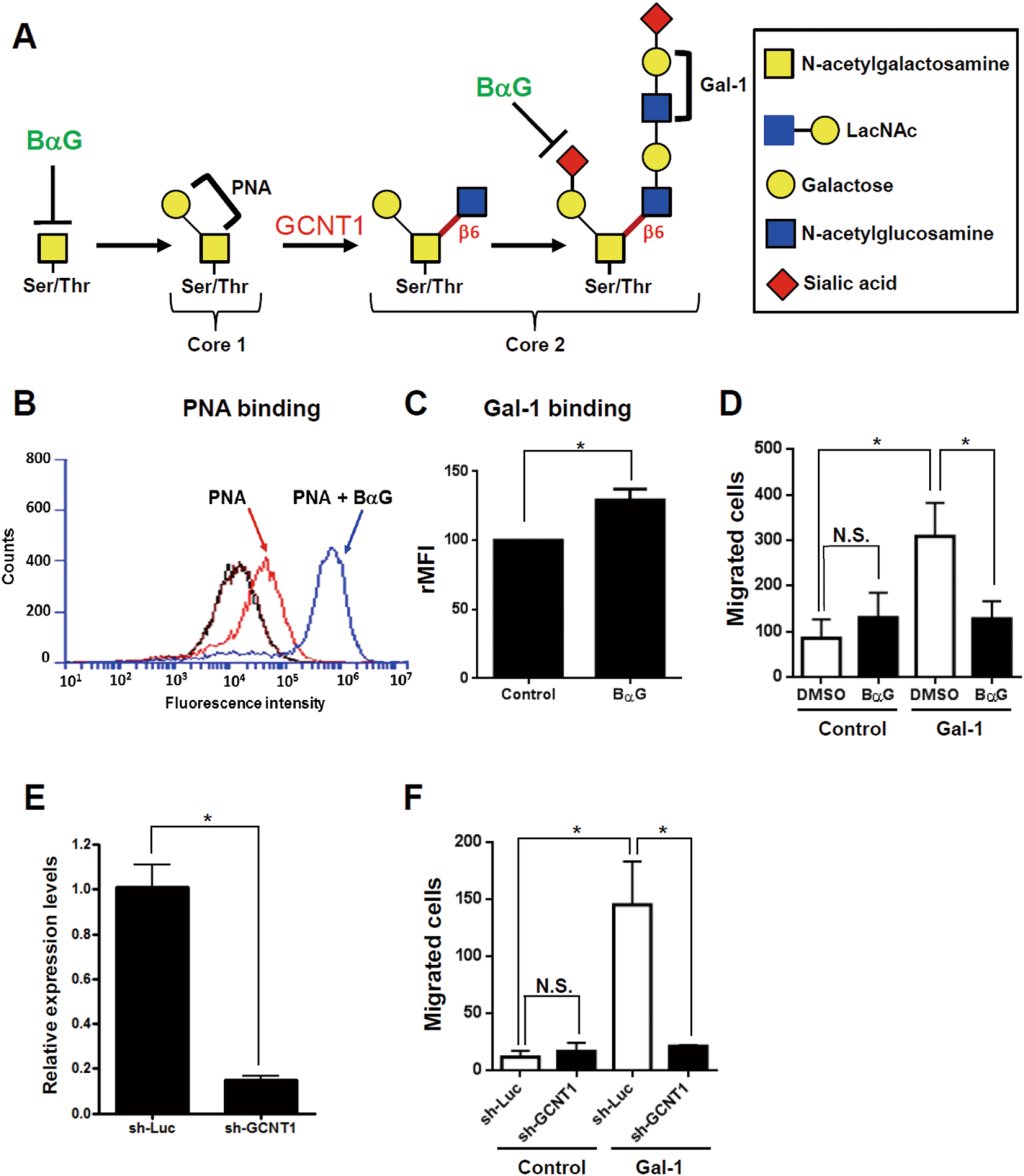
Blocking core 2 branching O-glycosylation suppressed Gal-1-induced hepatic stellate cell (HSC) migration. (**A**) Schematic representation of core 1 and core 2 O-glycan biosynthesis. (**B**) An O-glycan inhibitor (benzyl-N-acetyl-α-galactosaminide; BαG) increases PNA binding to LX-2 cells. (**C**) BαG increases the binding of Gal-1 and LX-2 cells. (**D**) BαG suppresses Gal-1-induced LX-2 migration. (**E**) Knockdown of core 2 N-acetylglucosaminyltransferase 1 (GCNT1) suppresses GCNT1 expression. (**F**) Knockdown of GCNT1 inhibits Gal-1-induced HSC migration. LX-2 cells were treated with BαG or infected with a lentivirus carrying GCNT1 shRNA for 48 h. After treatment, cells were incubated with PNA and Gal-1-488 to detect their binding to LX-2 cells using flow cytometry. To measure the migratory ability, cells were treated with 500 nM Gal-1 for 16 h. Migrated cells were counted, and results are presented as the mean ± SEM of three independent experiments. *p < 0.05.

The glycans present on the cell-surface were critical for Gal-1-induced HSC migration. Therefore, we characterized which glycan structure was critical for Gal-1 functions in activated HSCs by modifying N- and O-glycosylation. The biosynthesis of complex N-glycosylation is illustrated in Fig. 3A. Swainsonine (SW) was used to block poly-LacNAc elongation in complex N-glycans because it inhibits the activity of Golgi α-mannosidase II, a critical glycosidase for complex N-glycan formation (Fig. 3A). We found that SW treatment reduced the amount of L-PHA-reactive glycans (Fig. 3B) and accordingly, reduced Gal-1 binding and Gal-1-induced HSC migration (Fig. 3C,D). The results were further confirmed by knocking-down MGAT5 expression, which resulted in reduced L-PHA binding and MGAT5 expressions compared to control cells (Fig. 3E,F). Silencing MGAT5 also suppressed Gal-1-induced LX-2 cell migration (Fig. 3G) which demonstrated that complex N-glycans bearing poly-LacNAc glycans were required for Gal-1-induced migration of LX-2 cells. To investigate the role of O-glycan in Gal-1-induced HSC migration, we used benzyl-α-GalNAc (BαG) to modify O-glycan elongation. The biosynthesis of core 1 and core 2 O-glycans is illustrated in Fig. 4A. BαG treatment might change the O-glycan in two different ways: In cells with low-sialylated O-glycans, BαG blocks O-glycan elongation beyond the initial GalNAc residue, which can be detected by reduced PNA binding. PNA is a peanut agglutinin that recognizes core-1-galactosyl (β-1,3) *N*-acetylgalactosamine of O-glycan. In cells with high-sialylated O-glycans, BαG inhibits O-glycan sialylation which results in the exposure of nonsialylated O-glycans and increased PNA binding. In our results, BαG treatment increased PNA and Gal-1 binding in LX-2 cells (Fig. 4B,C), presumably due to increased exposure of nonsialylated core-1 ligands (low-affinity ligands) to Gal-1. However, BαG treatment decreased Gal-1-induced cell migration (Fig. 4D), which indicates that core 1 O-glycosylation is not required for Gal-1-induced HSC migration. However, we still did not know whether BαG suppressed Gal-1 induced HSC migration through blocking core-2 O-glycosylation. We thus examined whether core 2 branching poly-LacNAc is required for Gal-1-induced migration, because previous studies reported that GCNT1-mediated core 2 branching poly-LacNAc is critical for Gal-1 functions in cancer cells^20^ and T cells^21^. GCNT1 shRNA treatment decreased GCNT1 expression (Fig. 4E) and inhibited Gal-1-induced migration (Fig. 4F), thus confirming that core-2 branching poly-LacNAc glycans are required for Gal-1-induced HSC migration. In summary, the results indicated that both MGAT5- and GCNT1-mediated N- and O-glycan structures are critical for Gal-1-induced HSC migration.

### Glycosylation-dependent Gal-1 and NRP-1 interactions promote HSC migration

**Figure 5:**
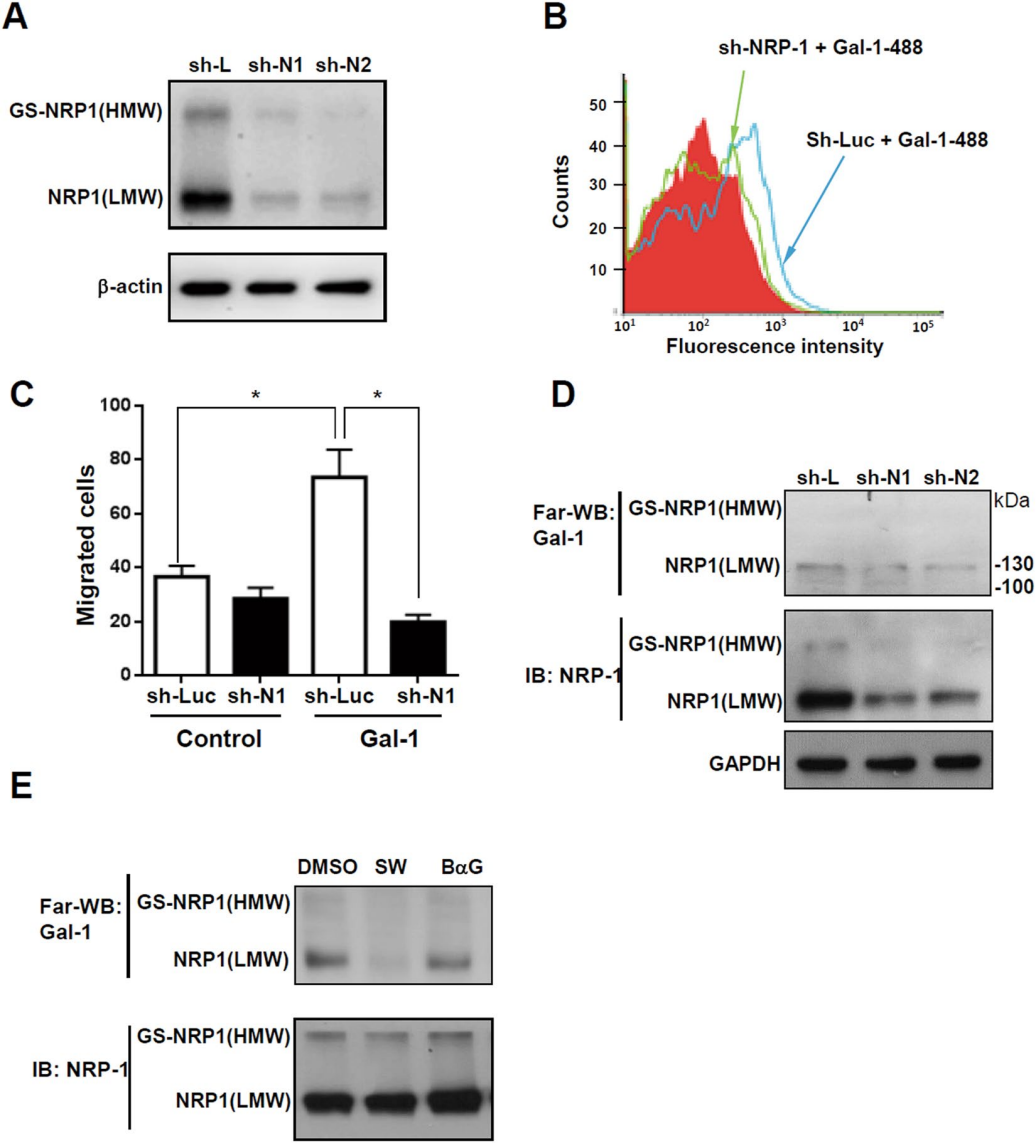
Glycosylation-dependent Gal-1/NRP-1 interactions induce hepatic stellate cell (HSC) migration. (**A**) NRP-1 shRNAs (sh-N1 and N2) suppress NRP-1 expression. (**B**) Knockdown of NRP-1 suppresses Gal-1 binding to LX-2 cells. (**C**) Knockdown of NRP-1 suppresses Gal-1-induced HSC migration. LX-2 cells were infected with a lentivirus carrying luciferase (sh-Luc) and NRP-1 shRNA (sh-N1). Gal-1 binding to LX-2 sh-Luc and sh-N1 cells was determined by flow cytometry. To measure the migratory ability, LX-2 sh-Luc and sh-N1 cells were treated with 500 nM Gal-1 for 16 h, and migrated cells were counted. Results are shown as the mean ± SEM of three independent experiments. *p < 0.05. (**D**) NRP-1 interacts with Gal-1. We performed the Far-Western blot in LX-2 cells transduced with luciferase (sh-L) and NRP-1 shRNAs (sh-N1 and N2) as described in “Materials and methods”. (**E**) Blocking N-glycosylation suppresses Gal-1 binding to NRP-1. The interaction between Gal-1 and NRP-1 was measured using Far-Western blotting as described in “Materials and methods”. LX-2 cells were treated with DMSO (vehicle), swainsonine (SW), and benzyl-N-acetyl-α-galactosaminide (BαG) for 48 h. The cell lysate was collected for Far-Western blotting.

We previously reported that NRP-1 is a critical glycosylated receptor of Gal-1 in endothelial cells and dermal fibroblasts^13, 17^, and recent studies demonstrated that NRP-1 is highly expressed in activated HSCs and regulates PDGF and TGF-β signaling^22, 23^. The evidence indicates that NRP-1 could be a glycosylated receptor for Gal-1 in HSCs. Therefore, we investigated whether N- and O-glycosylation of NRP-1 is required for Gal-1’s functions. Using a Western blot analysis, we found two isoforms of NRP1 in LX-2 cells, and both of their expressions could be suppressed by NRP-1 shRNA (Fig. 5A). A flow cytometric analysis revealed that knockdown of NRP-1 reduced Gal-1 binding to LX-2 cells (Fig. 5B) and significantly inhibited Gal-1-induced migration (Fig. 5C), indicating that NRP-1 is essential for Gal-1-induced HSC migration. To understand how glycosylation regulates the interaction between NRP-1 and Gal-1, LX-2 cells were treated with SW and BαG to modify the N- and O-glycan structures of NRP-1. The low-molecular-weight (LMW) NRP1 (~130 kDa) is presumably the N- and O-GalNAc (N-acetylgalactosamine) glycosylated NRP-1, and the high-molecular-weight (HMW) NRP-1 was presumed to be a glycosaminoglycan (GS) modification of NRP-1 as previously reported^24, 25^. Using Far-Western blotting, we found that one major band (~130 kD) interacted with Gal-1, presumably LMW NRP-1 and knockdown of NRP-1 suppressed the binding (Fig. 5D). Furthermore, we found that blocking the N-glycosylation of NRP-1 inhibited Gal-1 binding while blocking O-GalNAc glycosylation did not (Fig. 5E), which suggested that O-GalNAc glycosylation of other low-affinity receptors could be critical for Gal-1 induced migration and signaling because blocking the core-2 O-glycans was able to suppress Gal-1 induced migration (Fig. 4D). In summary, the results indicate that the N-glycosylated NRP-1 is the major receptor of Gal-1 and is critical for Gal-1-induced HSC migration. Although O-GalNAc glycosylation of NRP-1 is not required for Gal-1 binding, the O-GalNAc glycosylation of other low-affinity receptors, such as PDGF or TGF-β receptors (PDGFRs or TGF-βRs), may be required for Gal-1 induced HSC signaling and migration. It has been reported that galectin-3 (Gal-3) promotes HSC activation and liver fibrosis but Gal-3 did not interact with NRP-1 (Supplemental Fig. S5), suggesting Gal-3 regulates HSC homeostasis through distinct mechanisms compared to Gal-1.

### Galectin-1 induces TGF-β- and PDGF-like signaling through the NRP-1/PDGF receptor (PDGFR) and NRP-1/TGF-β receptor (TGF-βR) complex

**Figure 6:**
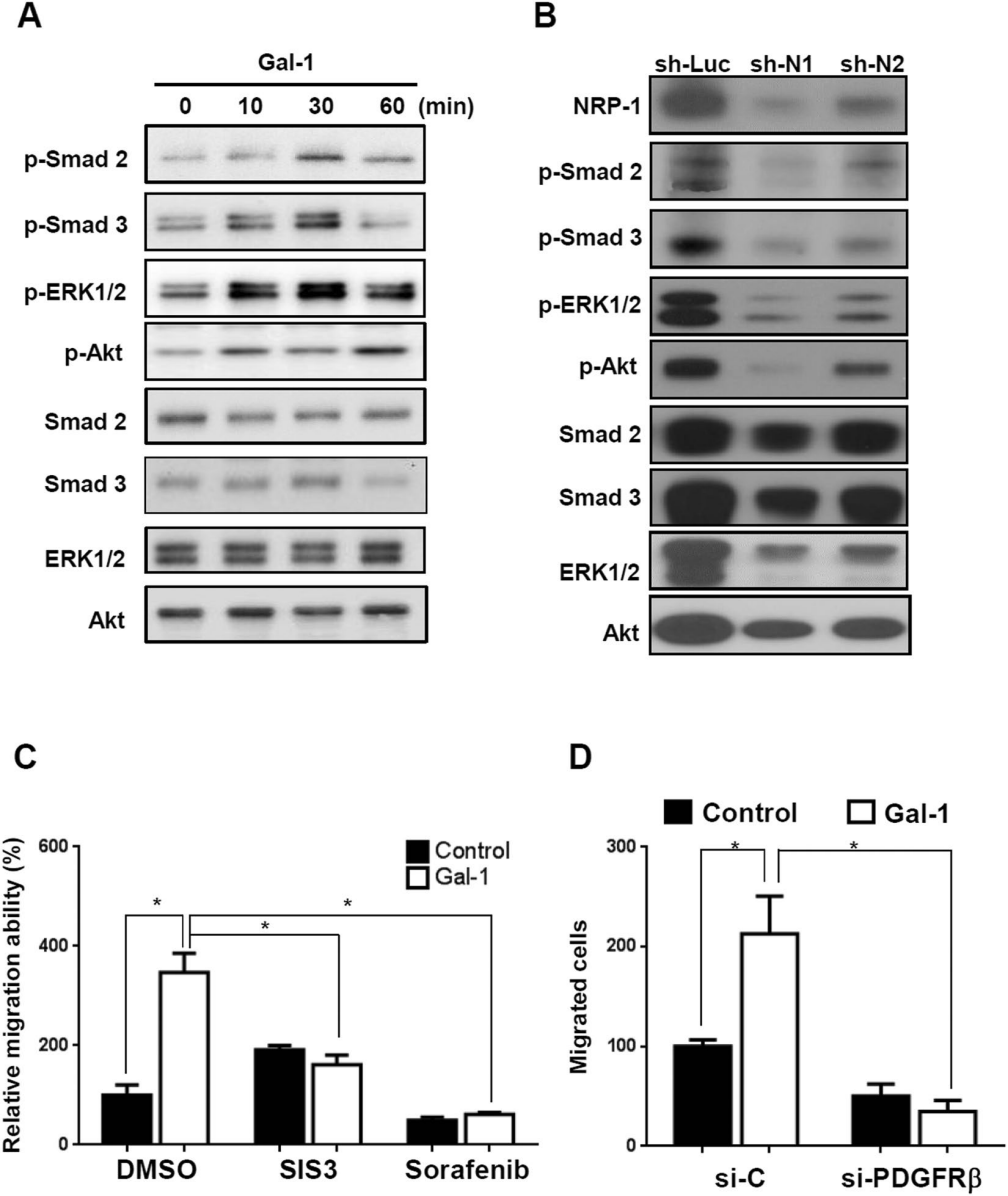
Gal-1 induces PDGF- and TGF-β-like signals through the NRP-1/PDGF receptor (PDGFR) and NRP-1/TGF-β receptor (TGF-βR) complex. (**A**) Gal-1 induced PDGF and TGF-β-like signaling in Gal-1 silencing cells (LX-2-shB09 cells). (**B**) Knockdown of NRP-1 suppressed Gal-1-induced signaling. LX-2 cells were serum-starved for 24 h followed by Gal-1 (500 nM) treatment for 10 min. The cell lysate was collected for Western blotting. (**C**) Sorafenib and SIS3 (a tyrosine kinase and a TGF-βR inhibitor) suppressed Gal-1-induced hepatic stellate cell (HSC) migration. For the migration assay, LX-2 cells were pretreated with sorafenib and SIS3 for 1 h. Then, cells were suspended and seeded into a transwell. After incubation for 24 h, migrated cells were counted, and results are presented as the mean ±SEM of three independent experiments. *p < 0.05. (**D**) Knockdown of PDGFRβ suppressed Gal-1-induced hepatic stellate cell (HSC) migration. LX-2 cells were transfected with control (si-C) and PDGFRβ siRNA (si-PDGFRβ). The cells were treated with Gal-1 and the migration ability was measured.

**Figure 7:**
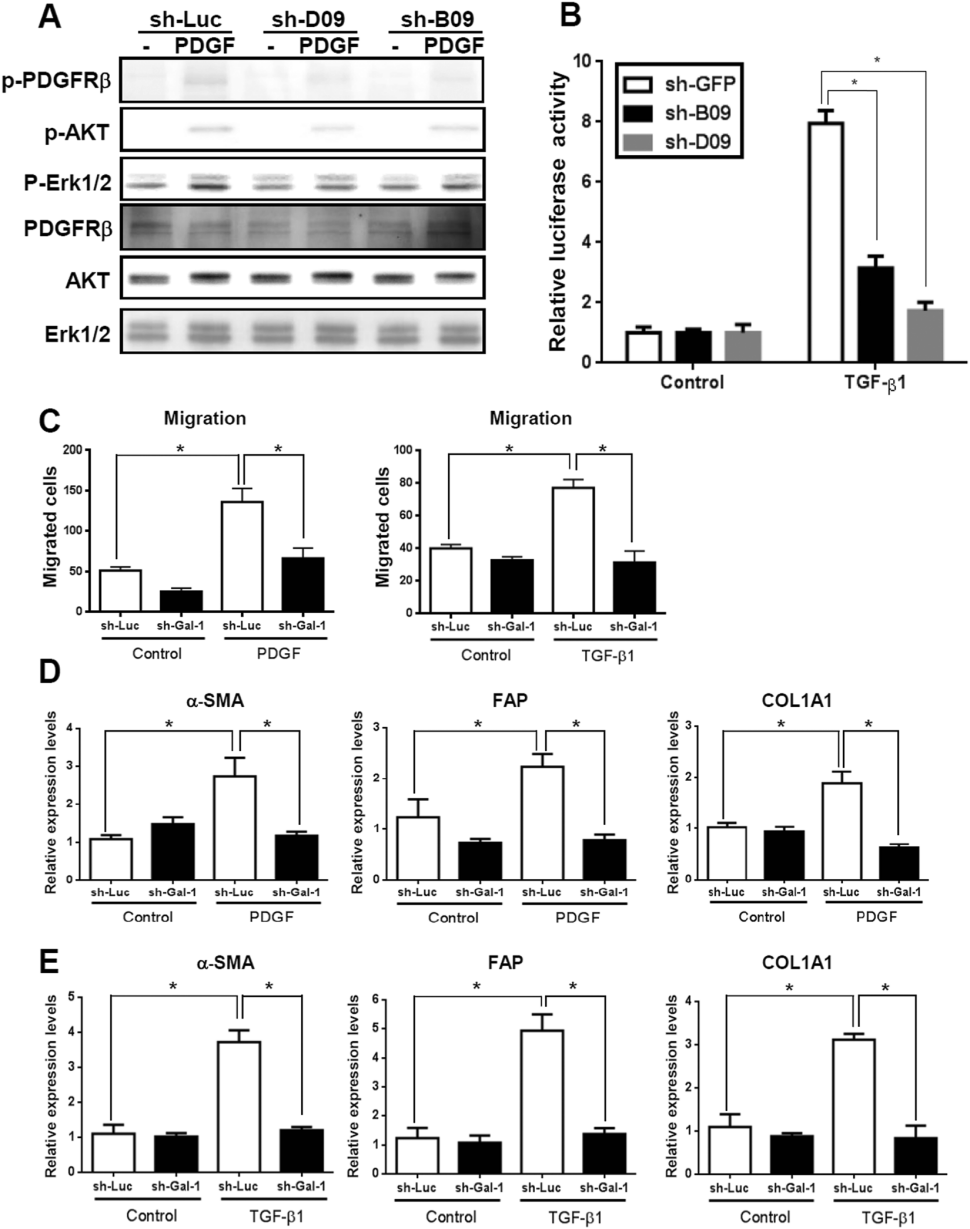
Knockdown of Gal-1 attenuates PDGF- and TGF-β-induced LX-2 cell signaling, gene expression, and migration. (**A**) Silencing Gal-1 suppresses PDGF-induced signaling. LX-2 cells were infected with a luciferase (sh-Luc) and Gal-1 shRNA (sh-B09, D09) lentivirus and were treated with PDGF for 10 min. The cellular phosphorylation of extracellular sign-regulated kinase 1/2 (Erk1/2), and Akt was measured using Western blotting. (**B**) Knockdown of Gal-1 suppresses the TGF-β-induced Smad2/3 transactivation ability. LX-2-sh-GFP, sh-B09, sh-D09 cells were transfected with a SBE4-Luc plasmid (luciferase reporter containing four copies of the Smad-binding site). Cells were starved for 24 h followed by TGF-β (1 ng/ml) treatment for 24 h, and luciferase activities were measured using the Luciferase Assay system (Promega). (**C**,**D**) Knockdown of Gal-1 inhibits PDGF- and TGF-β-induced gene expression. α-smooth muscle actin (α-SMA), fibroblast activation protein (FAP), and α-1 type I collagen (COL1A1) expressions in LX-2 cells were analyzed using RT-qPCR. Relative gene expression levels were calculated by comparing ∆CT values of each group to those of sh-Luc cells without treatment. Data are shown as folds of change. (**E**) Knockdown of Gal-1 expression inhibits PDGF- and TGF-β-induced HSC migration. Cell migration was measured using a Boyden chamber assay. Results are presented as the mean ± SEM of three independent experiments. *p < 0.05.

To understand how the Gal-1/NRP-1 axis influences HSC migration, we examined which signal was induced by Gal-1 stimulation. There is no kinase motif in the cytoplasmic domain of NRP-1, but it regulates intracellular signal transduction by forming a complex with PDGFRs or TGF-βRs in HSCs and non-HSC cells^22, 23, 26, 27^. We thus examined whether Gal-1 induces PDGF- and TGF-β-like signaling. Gal-1 treatment induced the phosphorylation of extracellular-regulated kinase 1/2 (Erk1/2), Akt, Smad 2, and Smad 3 (Fig. 6A), and the effect was blocked by NRP-1 shRNA (Fig. 6B). Furthermore, LX-2 cells were treated with sorafenib (a tyrosine kinase inhibitor) and SIS3 (a TGF-βR inhibitor) to block PDGF and TGF-β receptor activation. Both inhibitors suppressed Gal-1 induced HSC migration (Fig. 6C), indicating that both PDGFR and TGF-βR activation are required for Gal-1-induced HSC migration. Because sorafenib may also inhibit molecular targets parallel to PDGFRs, we used PDGFRβ siRNA to specifically block PDGFR signaling, and found that Gal-1 induced HSC migration was decreased (Fig. 6D). In addition, because it is known that Gal-1 induces co-clustering of membrane receptors that potentiates extracellular growth factor or inflammatory stimulation^28^, we examined whether Gal-1 regulates TGF-β- and PDGF-induced signaling. Knockdown of endogenous Gal-1 in LX-2 cells reduced PDGF-induced signal transduction (Fig. 7A), and suppressed TGF-β-induced SBE4-luciferase promoter activity (luciferase reporter containing four copies of the Smad-binding site) (Fig. 7B). Knockdown of Gal-1 also inhibited PDGF- and TGF-β-induced HSC migration (Fig. 7C) and gene expressions such as α-SMA, FAP, and COL1A1 (Fig. 7D,E). In summary, these results indicate that Gal-1 not only induces PDGF- and TGF-β-like signals through NRP-1 in HSCs but also potentiates TGF-β- and PDGF-induced HSC activation and migration.

### Loss of Gal-1 attenuates methionine- and choline-deficient diet (MCD)-induced liver fibrosis

**Figure 8:**
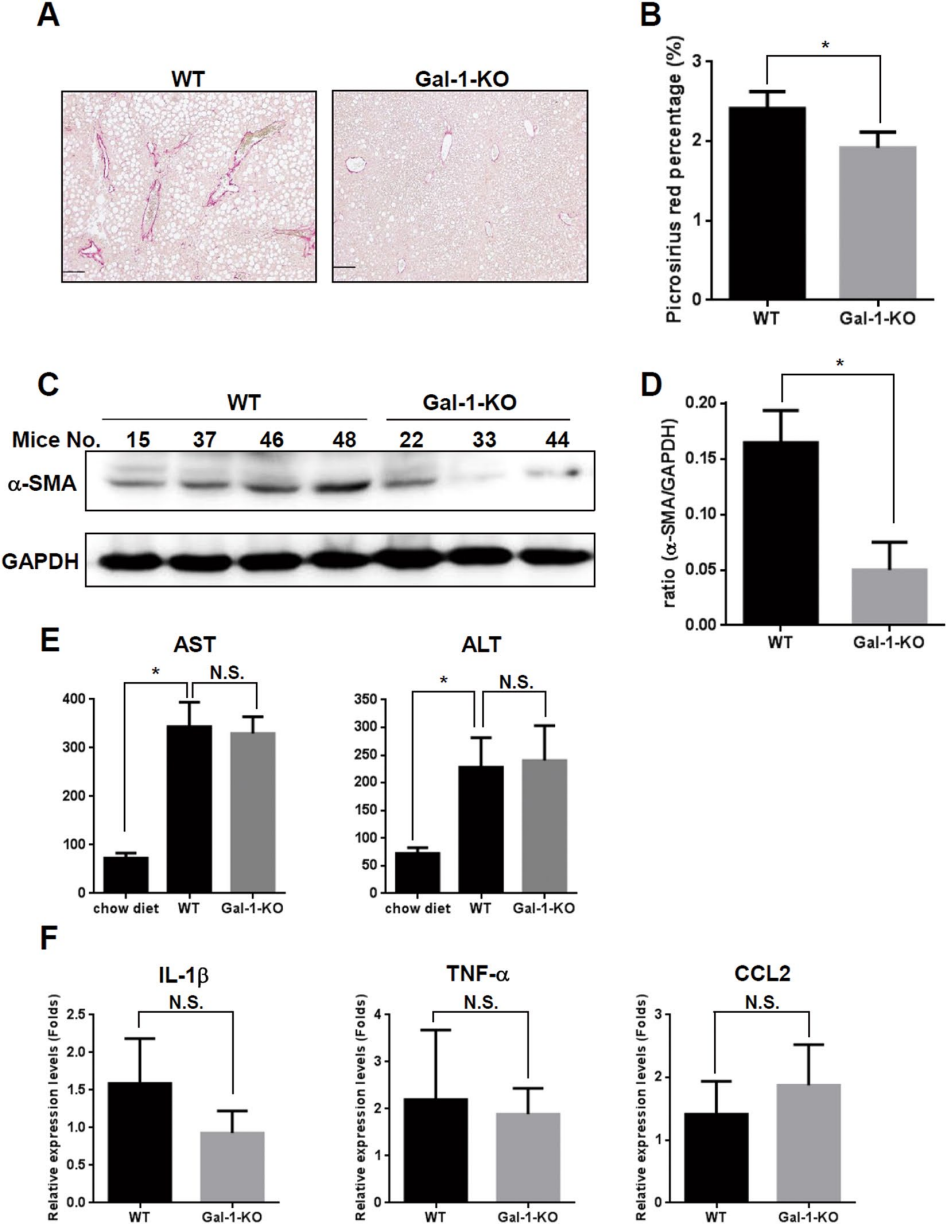
Loss of Gal-1 suppresses methionine- and choline-deficient (MCD) diet-induced liver fibrosis. (**A**,**B**) MCD diet-induced collagen deposition is reduced in Gal-1 null mice (Gal-1-KO) (n = 6) compared to wild-type mice (WT) (n = 6). Liver fibrosis was induced by feeding mice with an MCD diet for 8 weeks. Collagen was detected using picrosirius red staining. The left panel shows the representative images. The right panel shows the quantitative data of picrosirius red staining using ImageJ software. (**C**,**D**) HSC activation is reduced in Gal-1 null mice compared to wild-type mice. HSC activation was examined by measuring α-smooth muscle actin (α-SMA) expression using Western blotting (left panels) which  confirmed that loss of Gal-1 suppresses HSC activation and extracellular matrix production in livers of MCD diet-fed mice. (**E**) Serum AST and ALT amount are similar between Gal-1 null mice and wild-type mice. Serum AST and ALT were measured using a VetTest® Chemistry Analyzer. (**F**) Loss of Gal-1 does not change the RNA expression levels of proinflammatory cytokines (IL-1β, TNF-α, CCL2) compared to wild-type mice. The RNA was extracted from mouse livers and was converted to cDNA. Proinflammatory cytokines expression was measured using RT-qPCR. Relative expression levels were calculated by comparing ∆CT values of each group to those of wild-type mice without treatment. Data are shown as folds of change.

To examine the functional relevance of Gal-1-induced HSC migration and activation *in vivo*, liver fibrosis was induced in mice by feeding them an MCD for 8 weeks^29^. Picrosirius red and Masson’s trichrome staining revealed reduced collagen deposition in Gal-1-null mice compared to wild-type mice (Fig. 8A,B and Supplemental Fig. S6). Western blotting also showed a decreased α-SMA expression in Gal-1-null mice compared to wild-type mice. However, the amount of pro-inflammatory cytokine, serum AST (aspartate aminotransferase) and ALT (alanine transaminase) were similar between Gal-1 KO and wild-type mice feed with MCD diet (Fig. 8E,F). We thus examined whether the macrophages express Gal-1 in MCD-fed mice using anti-CD68 (a macrophage marker) and anti-Gal-1 antibodies. The CD68 staining was co-localized with Gal-1 indicating that macrophages expressed Gal-1 (Supplemental Fig. S7). However, there were few CD68-positive signals in the livers indicating the MCD feeding did not induce strong inflammatory responses in the livers. Therefore, it is suggested that Gal-1 mainly affects fibrogenesis in the MCD-fed mice, and the inflammatory effect of Gal-1 may not be significantly detected because of the mild inflammation in this model.

## Discussion

**Figure 9:**
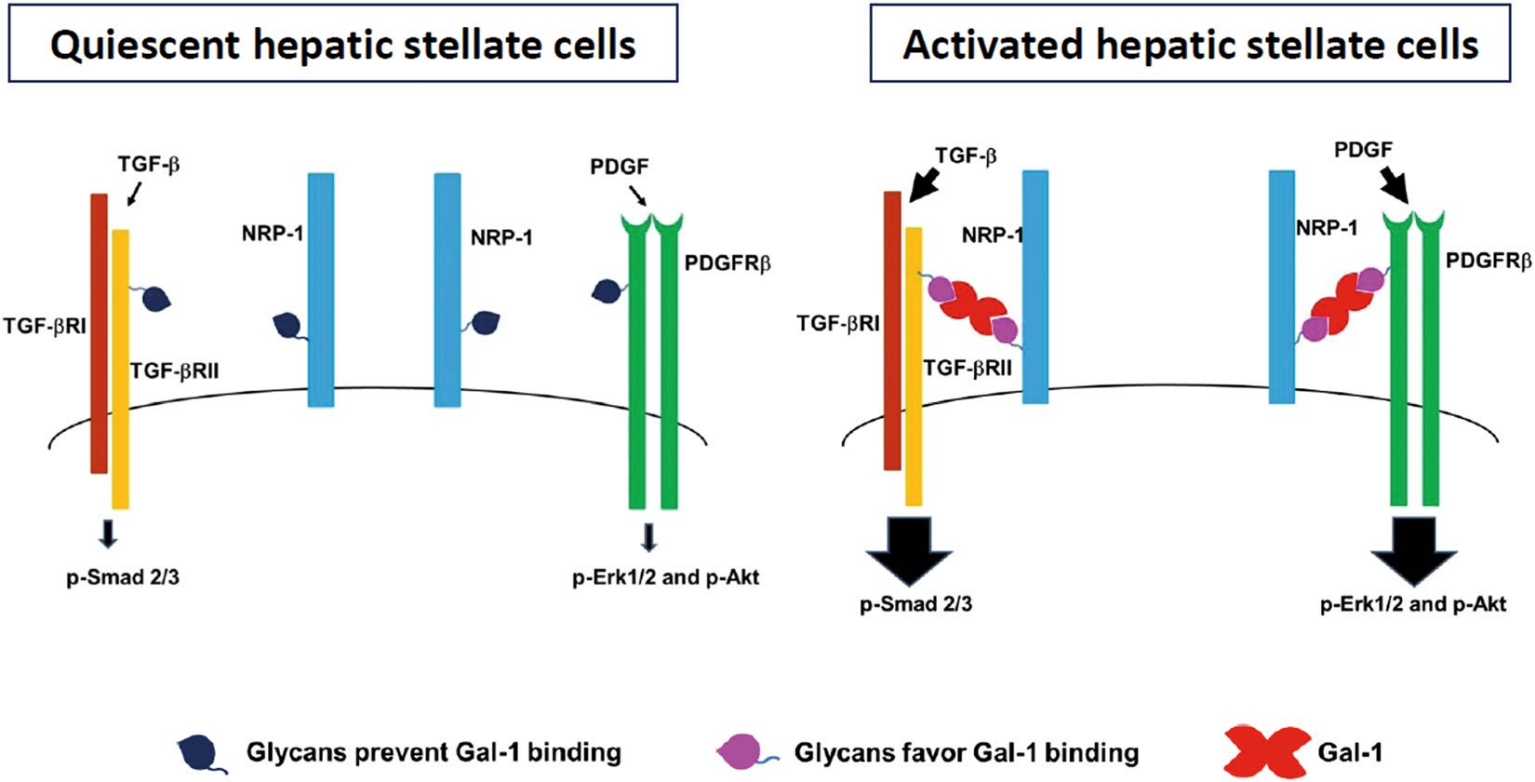
A proposed model: the co-evolution of the HSC glycome and Gal-1 promotes HSC activation and migration. First, Gal-1 is highly expressed in activated HSCs compared to quiescent HSCs. Second, the “Gal-1-permissive” glycan repertoire (greater poly-LacNAc-modified glycans and less terminal sialic acid modification) in activated HSCs facilitates Gal-1 to induce PDGF- and TGF-β-like signals through the co-clustering of NRP-1/PDGFRs and NRP-1/TGF-βRs. In contrast, the “Gal-1 non-permissive” glycan repertoire in quiescent HSCs provides low-affinity ligands for Gal-1 binding, which induces weak PDGF and TGF-β signals.

Previous studies reported glycosylation alterations in HSC activation and liver fibrosis using high-throughput analyses. For example, using an oligonucleotide and lectin microarray, Yu *et al*. reported that both glycan-modification gene and glycan profiles changed in fibrotic livers compared to normal livers^30^. Mondal *et al*. reported distinct glycosylation of serum proteins in HBV- and HCV-related cirrhotic patients using 2D gel electrophoresis and liquid chromatography-mass spectroscopy (LC-MS)^31^. However, it is unclear whether altered glycosylation affects HSC homeostasis and liver fibrosis. In this study, we highlighted the coordinated effect of cell-surface glycans and Gal-1 on HSC homeostasis and integrated glycosylation-dependent Gal-1/NRP-1 interactions into canonical PDGF- and TGF-β-induced signaling pathways. Based on the results, we proposed a model in which co-evolution of the HSC glycome and Gal-1 promotes HSC activation and migration (Fig. 9). First, Gal-1 is highly expressed in activated HSCs compared to quiescent HSCs. Second, the “Gal-1-permissive” glycan repertoire (greater poly-LacNAc-modified glycans and less terminal sialic acid modification) in activated HSCs facilitates Gal-1 to induce PDGF- and TGF-β-like signals through the co-clustering of NRP-1/PDGFRs and NRP-1/TGF-βRs. In contrast, the “Gal-1 non-permissive” glycan repertoire in quiescent HSCs provides low-affinity ligands for Gal-1 binding, which induces weak PDGF and TGF-β signals. Our results not only demonstrated the cooperative effect of Gal-1 and glycosylation changes in HSC activation but also implied that targeting glycosylation-dependent Gal-1/NRP-1 interactions might be effective in liver fibrosis therapy. In agreement with this hypothesis, adenoviral vector-mediated MGAT5 silencing, which reduced the glycan ligands for galectins, ameliorated hepatoxin-induced liver fibrosis^32^. In addition, although we proposed that Gal-1 induces a co-clustering of NRP-1/PDGFRs and NRP-1/TGF-βRs, Gal-1 may alternatively bind to the NRP-1/PDGFRs and NRP-1/TGF-βRs complexes. The additive or synergic effect of Gal-1, PDGF, and TGF-β may further enhance HSC activation and the progression of liver fibrosis.

We found that Gal-1 interacts with NRP-1 in an N-glycosylation-dependent manner which is consistent with a previous finding^33^. However, the glycosylation status of NRP-1 is poorly understood. Several N- and O-glycosylation sites of NRP-1 has been identified^24, 34–36^, but there are few publications addressing the biological effects of N- and O- glycosylation on NRP-1’s functions. Glycosaminoglycan (GAG) modification was the best-characterized O-glycosylation of NRP-1, which is characterized by heparin or chondroitin attached to ser^612^ (refs 24 and 25) and a HMW NRP-1 (>180 kDa). GAG-modified NRP-1 regulated VEGF- and PDGF-induced intracellular signaling and biological functions in smooth muscle cells^24, 37^ and glioblastoma cells^25^. However, in our results, Gal-1 interacted with the LMW NRP-1 (~130 kDa) instead of GAG-modified NRP-1. Furthermore, blocking N-glycosylation reduced Gal-1/NRP-1 interactions and suppressed Gal-1-induced HSC migration, which highlights the critical role of N-glycosylation in modulating NRP-1’s functions. Meanwhile, we found that O-GalNAc glycosylation of NRP-1 was not required for Gal-1 binding but blocking core-2 O-glycosylation suppresses Gal-1 induced migration. The contradictory results may be explained by that O-GalNAc glycosylated PDGFRs and TGF-βRs are low-affinity receptors for Gal-1 but they are required for signal transduction. Furthermore, since it has been known that NRP-1 forms a complex with PDGFRs and TGF-βRs in HSCs^22, 23^, we proposed the N-glycan of NRP-1 is responsive for Gal-1 binding and the O-GalNAc glycosylation of PDGFRs and TGF-βRs are responsive for Gal-1 to transduce signaling. Interestingly, another study proposed that GAG-modified NRP-1 increased in activated HSCs, which forms a “molecular net” to sequester local growth factors such as PDGF and TGF-β^22^. Therefore, these results indicated that both GAG and N-glycosylation of NRP-1 could influence PDGF- and TGF-β-induced HSC activation and migration. Full characterization of NRP-1 glycan structures in HSCs will be important to understand the mode of the Gal-1/NRP-1 interaction and its role in HSC activation and migration.

Because members of the galectin family recognize galactoside, it was reported that galectins have redundant roles in regulating biological functions such as plasma cell formation^38^, tumor angiogenesis^39^, T cell apoptosis^40^, and wound healing^41^. Intriguingly, previous studies also reported that Gal-3 regulates rat HSC proliferation^42^ and activation, and liver fibrosis^43^. Therefore, the glycan repertoire of activated HSCs may facilitate Gal-3 binding, which explains why there is a redundancy of Gal-1 and Gal-3 in HSC activation and proliferation. However, we speculated that Gal-1 and Gal-3 stimulate partly distinct signaling pathways in HSCs, because a microarray analysis of Gal-1- and Gal-3-knockdown HSCs showed distinct gene expression profiles (our unpublished data). Differences may have resulted from the distinct protein structures of Gal-1 and Gal-3, which may selectively facilitate their recognition of distinct glycoproteins. For instance, we found that Gal-1 binds to NRP-1 but Gal-3 does not (Supplemental Fig. S4). Recently, two galectin inhibitors, GR-MD-02 and GM-CT-01, showed therapeutic effects on thioacetamide (TAA)- and non-alcoholic steatohepatitis (NASH)-induced liver fibrosis^44, 45^, but the underlying mechanisms are largely unknown. Although both GR-MD-02 and GM-CT-01 can bind Gal-1 and Gal-3^45^, the authors speculated that Gal-3 is the major target of GR-MD-02 and GM-CT-01, because Gal-3-null mice were protected from CCl_4_-induced liver fibrosis^43^ and high-fat diet-induced NASH^46^. Our results provide an alternative mechanism for the two galectin inhibitors that target Gal-1 in HSC activation and migration. Regardless of which pathways of Gal-1 and Gal-3 play a role in HSCs, results from the current study demonstrate that targeting either Gal-1 or Gal-3 can lead to effective results in suppressing HSC activation and liver fibrosis. Dissecting the convergent and distinct roles of Gal-1 and Gal-3 in liver fibrosis using Gal-1, Gal-3, and Gal-1/Gal-3 double-knockout mice will benefit the development of anti-galectin drugs for liver fibrosis.

In summary, we discovered a novel mechanism through which concomitant expressions of cell-surface glycans and Gal-1 promote HSC activation and migration through the NRP-1/PDGFRβ and NRP-1/TGF-βRII complex. These findings provide new implications for the development of liver fibrosis therapy by disrupting glycosylation-dependent Gal-1/NRP-1 interactions.

## Materials and Methods

### Cells and reagents

LX-2 cells, an immortalized hepatic stellate cell line^47^, were cultured in Dulbecco’s modified Eagle medium (DMEM) plus 10% fetal bovine serum (FBS) which was kindly provided by Dr. Yi-Tsau Huang (National Yang-Ming University, Taipei, Taiwan). Antibodies including anti-p-Erk1/2, anti-p-Akt, anti-Erk1/2, anti-Akt, anti-p-smad2, anti-p-smad3, anti-smad2, and anti-smad3 were from Cell Signaling. Anti-neuropilin (NRP)-1 and α-smooth muscle actin (α-SMA) antibodies were from GeneTex and Millipore. The polyclonal anti-Gal-1 antibody was purified from Gal-1-immunized rabbit serum. Recombinant PDGF and TGF-β were from R&D Systems. Inhibitors of N- and O-glycosylation [swainsonine (SW) and benzyl-N-acetyl-α-galactosaminide (BαG)] were from Calbiochem. Sorafenib and SIS3 were from MedChemexpress (MCE). Biotinylated lectins including L-PHA, LEL, SNA, PNA and DyLight® 488 streptavidin were from Vector Labs. Thiodigalactoside (TDG) was from Carbosynth.

### Animal models

Gal-1 null mice were originally produced and deposited in the Mutant Mouse Regional Resource Center by the Consortium for Functional Glycomics supported by the National Institute of General Medical Sciences (GM62116, Poirier and Robertson, 1993). The animals were raised and cared for according to the guidelines of the National Science Council, Taiwan. Liver fibrosis was induced in 6~8-week-old C57BL/6 mice by an intraperitoneal (IP) injection of thioacetamide (TAA, 200 mg/kg) or carbon chloride (CCl_4_, 1 ml/kg diluted 1:1 in olive oil) twice a week or by feeding mice a methionine- and choline-deficient diet (MCD). After 8 weeks of injections or feeding, the mice were sacrificed. The liver and whole body weights were measured, and RNA and protein samples were extracted from the first lobe of the livers. Other parts were fixed in formalin for immunostaining. The protocol was approved by the institutional animal care and use committee of Taipei Medical University (approval number:LAC-2013-0291) and carried out under the institutional and the ARRIVE guidelines (https://www.nc3rs.org.uk/arrive-guidelines) with animal welfare standards.

### Cell migration assay

Cell migration assays were done in 24-well transwell polycarbonate filters (pore size, 8 mm; Corning Costar). Briefly, LX-2 cells were seeded in the upper chamber, and chemoattractants such as Gal-1, PDGF, and TGF-β were added to the lower chamber. Migrated cells were counted after cells had been individually incubated for 24 h. Non-penetrating cells were removed from the upper surface of the filter with a cotton swab. Penetrating cells were fixed and stained with a DiffQuick stain kit (Dade Behring) according to the manufacturer’s instructions. For quantification, all of the cells that had migrated onto the lower surface were stained and counted under a light microscope. All experiments were performed in duplicate. Results are shown as the mean ± standard error of the mean (SEM) of three independent assays.

### Quantitative real-time polymerase chain reaction (RT-qPCR)

Total RNA was isolated using a Trizol reagent (Invitrogen), and then 1 µg was reverse-transcribed. Expressions of different messenger (m)RNAs were measured with an RT-qPCR as previously described^12^.

### Small hairpin (sh)RNA lentivirus production

Lentiviral vectors containing luciferase, green fluorescence protein (GFP), Gal-1, NRP-1, and core 2 N-acetylglucosaminyltransferase 1 (GCNT1) shRNAs were obtained from the National RNAi Core Facility (Academia Sinica, Taipei, Taiwan) and prepared in accordance with standard protocols. Briefly, 3 × 10^6^ 293T cells were seeded in a 10-cm dish. After a 24-h incubation, cells were co-transfected with 5 µg pLKO.1 shRNA, 5 µg pCMVΔR8.91, and 0.5 µg pMD.G plasmids by Lipofectamine 2000 (Invitrogen). After transfection, the transfection reagents were removed, and new medium was added. The virus was collected 48 h later and filtered through 0.45-mm filters. Cells were infected with a lentivirus (at a multiplicity of infection of 1) in the presence of polybrene (8 mg/mL). Forty-eight hours post-infection, cells were treated with 1 µg/mL of puromycin to select puromycin-resistant clones. The target sequences are listed below: Gal-1(B09): GCTGCCAGATGGATACGAATT; Gal-1(D09): CGCTAAGAGCTTCGTGCTGAA; NRP-1(N1): CCGAGAGAACAAGGTGTTCAT; NRP-1(N2): CAGCCTTGAATGCACTTATAT; GCNT1: GCGGAGTTTCCAATAGCATAT.

### Short interfering (si)RNA transfection

MGAT5 expression was silenced by siRNA transfection using Lipofectamine 2000 (Invitrogen) according to the manufacturer’s instructions. MGAT5 and control siRNAs were synthesized by GenePharma. Sequences of MGAT5 were 5′-GGCGGAAAUUCGUACAGAUTTAUCUGUACGAAUUUCCGCCTT-3′ (MGAT5-siRNA1) and 5′-CCUGGAAGCUAUCGCAAAUTTAUUUGCGAUAGCUUCCAGGTT-3′ (MGAT5-siRNA3). The control siRNA sequence was 5′-UUCUCCGAACGUGUCACGUTTACGUGACACGUUCGGAGAATT-3′.

### Immunohistochemical (IHC) and immunofluorescence analysis

Serial 5-µm histological sections were deparaffinized in xylene and rehydrated. After blocking of endogenous peroxidase by incubation with 3% hydrogen peroxide, slides were incubated with anti-Gal-1 and anti-α-SMA antibodies overnight at 4 °C, and the signal was visualized by applying the PolyDetector HRP Detection System (Bio-SB). Slides were counterstained with hematoxylin and mounted with mounting solution. Picrosirius red and Masson’s trichrome stains were used to visualize collagen using two kits (Polysciences and Sigma). For immunofluorescence analysis, the slides were incubated with anti-Gal-1, anti-CD68 and anti-α-SMA antibodies overnight at 4 °C, and the signal was visualized by Alexa Fluor-488 and -594 secondary antibodies. For L-PHA binding, the slides were incubated with biotinylated L-PHA for 30 min followed by DyLight® 488 streptavidin staining for 30 min. The slides were mounted with DAPI solution and were visualized using a fluorescence microscopy.

### Luciferase assay

LX-2 cells (2 × 10^5^) were seeded in 24-well dishes and were transfected with 0.4 µg of the SBE4-Luc plasmid (luciferase reporter containing four copies of the Smad-binding site), and 0.1 µg of the pCMV-LacZ plasmid (Clontech) was co-transfected in all transfection experiments as an internal control. Cells were starved for 24 h followed by TGF-β (1 ng/ml) treatment for 24 h, and luciferase activities were measured with the Luciferase Assay system (Promega). All luciferase activities were normalized to β-galactosidase activity.

### Far-Western blotting

Cell lysates (500 µg) were pre-cleaned by incubation with 2 µg of rabbit immunoglobulin G (IgG) overnight under agitation and 50 µl of protein A/G-coupled Sepharose beads (Santa Cruz Biotechnology) was added to the tube and incubated for 2 h at 4 °C. After incubation, the supernatant was collected and boiled at 95~100 °C for 5 min for sodium dodecylsulfate polyacrylamide gel electrophoresis (SDS-PAGE). Proteins were transferred to polyvinylidene difluoride membranes, and incubated with the Gal-1 recombinant protein (500 nM) for 16 h. Membranes were probed with an anti-Gal-1 antibody followed by anti-rabbit IgG conjugated with horseradish peroxidase (HRP). The NRP1/Gal-1 complex was visualized by adding enhanced chemiluminescence (ECL) substrate to the membrane.

### Flow cytometric analysis

Cells (10^5^) were suspended in phosphate-buffered saline (PBS) and incubated with different types of biotinylated lectins including L-PHA (2 μg/ml), LEL (1 μg/ml), SNA (5 μg/ml), and PNA (10 μg/ml) for 30 min followed by DyLight® 488 streptavidin staining for 30 min. For the Gal-1-binding analysis, recombinant Gal-1 was labeled with a DyLight labeling kit (Thermo Scientific) following the manufacturer’s instructions, and 10^5^ cells were incubated with fluorescence-labeled Gal-1 (Gal-1-488, 10 μg/ml) for 30 min. Cells were analyzed using a BD Accuri™ C6 Cytometer (BD Biosciences).

### Statistical analysis

All experiments were performed in duplicate. Results are shown as the mean ±SEM of three independent assays. Results were assessed using an unpaired Student’s *t*-test or 1-way analysis of variance (ANOVA). Statistical significance was set at *p* < 0.05.

## Electronic supplementary material

Supplemental information
