# Supplementary material for: Glycosylation-dependent galectin-1/neuropilin-1 interactions promote liver fibrosis through activation of TGF-β- and PDGF-like signals in hepatic stellate cells
Source: Sci Rep. 2017 Sep 8;7:11006. doi: 10.1038/s41598-017-11212-1 (PMC5591297; doi:10.1038/s41598-017-11212-1)

Supplemental information

Glycosylation-dependent galectin-1/neuropilin-1 interactions promote liver fibrosis through activation of TGF-β- and PDGF-like signals in hepatic stellate cells

Ming-Heng Wu[1,7*], Yuh-Ling Chen[2], Kuen-Haur Lee[3], Che-Chang Chang[1], Tsai-Mu Cheng[1], Szu-Yuan Wu[4,5], Chao-Chiang Tu[6], and Wan-Lin Tsui[1]

[1]*Graduate Institute of Translational Medicine,* [3]*Graduate Institute of Cancer Biology and Drug Discovery, College of Medical Sciences and Technology, Taipei Medical University, Taipei, Taiwan*

[2]*Institute of Oral Medicine, College of Medicine, National Cheng Kung University, Tainan, Taiwan*

[4]*Department of Radiation Oncology, Wan Fang Hospital, Taipei Medical University, Taipei, Taiwan*

[5]*Department of Internal Medicine, School of Medicine, College of Medicine, Taipei Medical University, Taipei, Taiwan*

[6]*Department of General Surgery, Taipei Medical University Hospital, Taipei Medical University, Taipei, Taiwan*

[7]*Center for Cell Therapy and Regeneration Medicine, Taipei Medical University, Taipei, Taiwan*

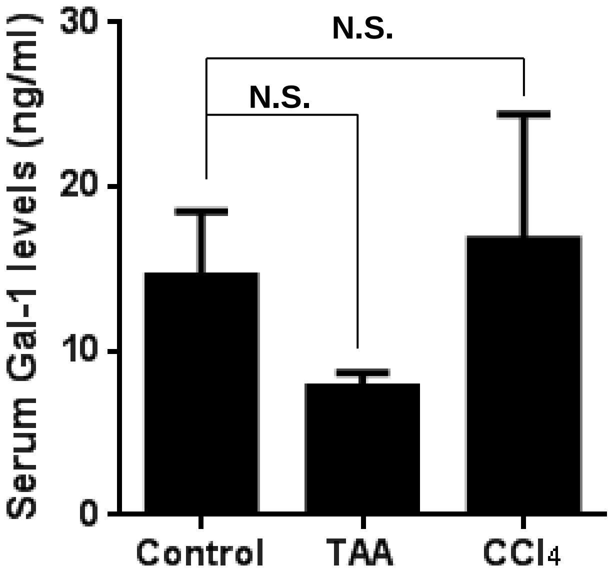

Figure S1. There is no significant difference of serum galectin-1 (Gal-1) levels among the control (n=3), TAA (n=4) and CCl_4 (n=4)-treated mice. Mouse serum Gal-1 was examined using a Mouse Galectin-1 ELISA Kit (Abcam, ab119595) as the manufacture's instructions. N.S. no significant difference.

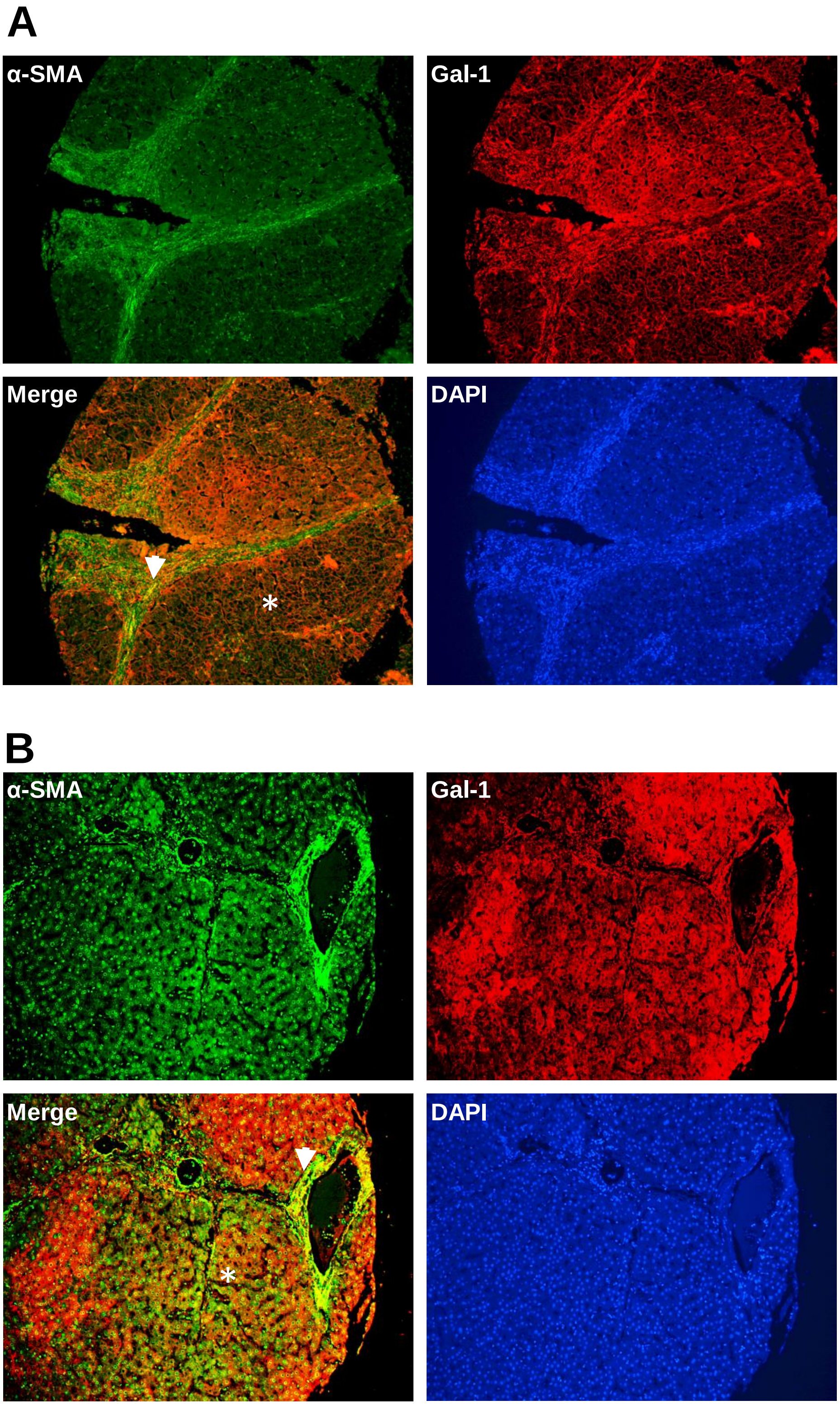

Figure S2. Immunofluorescence (IF) staining of human cirrhotic tissues. IF staining was performed as described in “Materials and methods”. **(A)** Gal-1 and α-SMA are up-regulated in non-parenchymal regions. **(B)** Gal-1 and α-SMA are up-regulated in both non-parenchymal and parenchymal regions. Asterisks indicate parenchymal cells, and arrowheads indicate non-parenchymal cells.

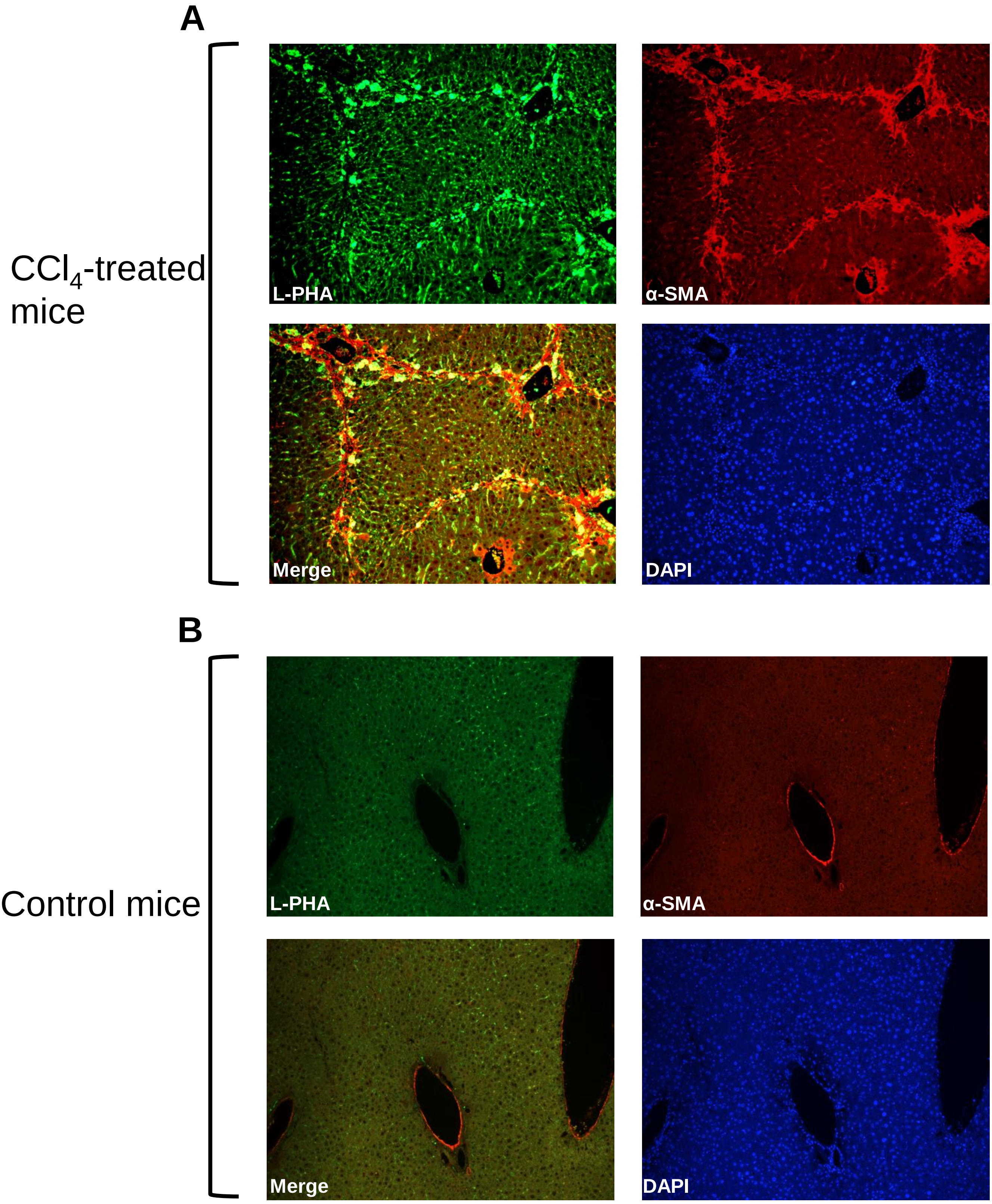

Figure S3. Immunofluorescence (IF) staining of the livers of CCl_4-treated **(A)** and control mice **(B)**. IF staining was performed as described in "Materials and methods". L-PHA binding and α-SMA expression are up-regulated in the livers of CCl4-treated mice compared to control mice.

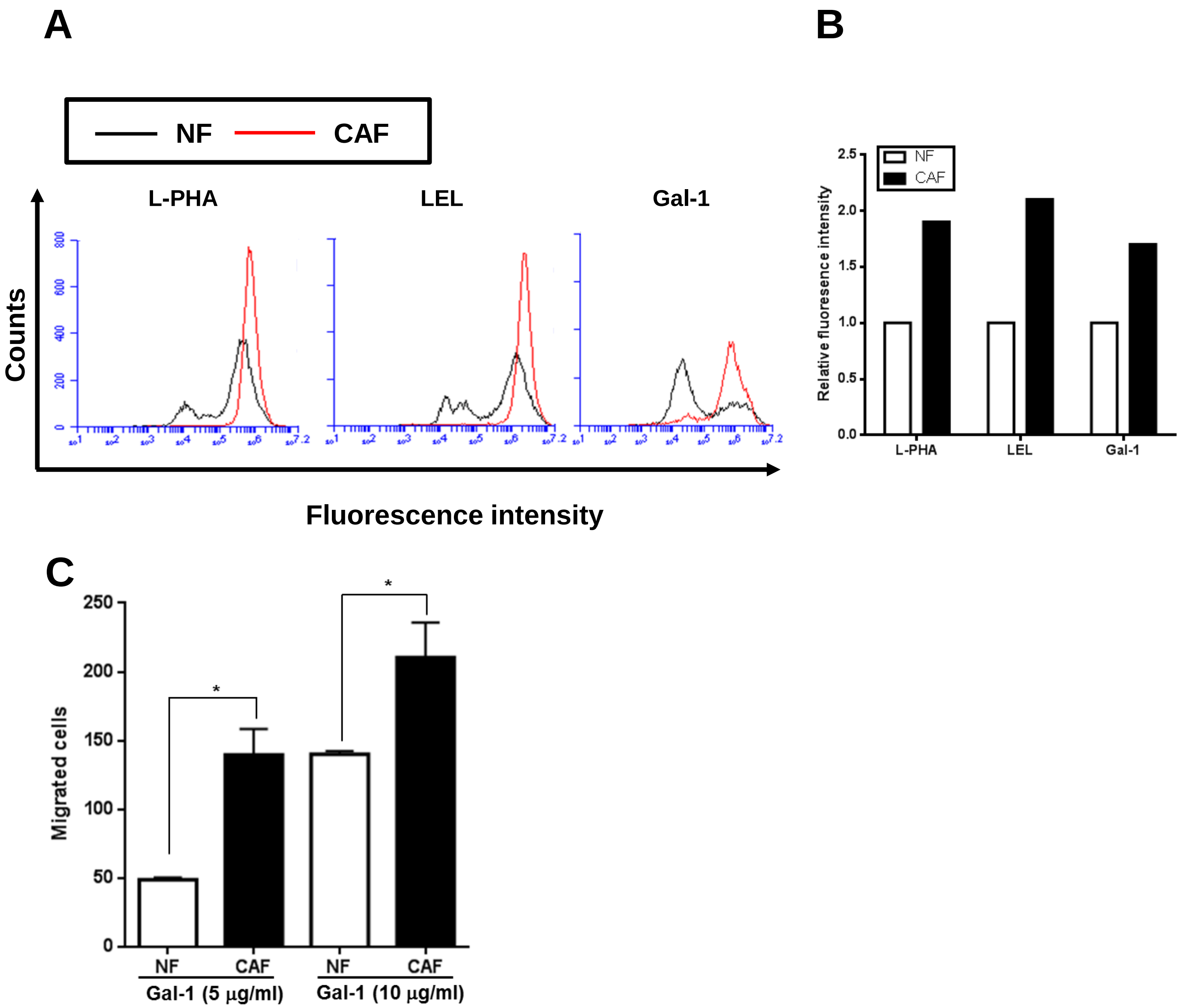

Figure S4. (A) Carcinoma-associated fibroblasts (CAFs) have higher amounts of β1,6 GlcNAc-branched N-glycans (L-PHA binding), and poly-N-acetylactosamine structures (LEL binding) compared to normal fibroblasts (NFs). NFs and CAFs were incubated with different lectins (L-PHA, LEL, and Gal-1), and lectin binding was analyzed by flow cytometry. **(B)** Quantitation of the relative fluorescence intensity between NFs and CAFs. The rMFI was calculated by comparing the mean fluorescence intensity of CAFs to that of NFs, and results are shown as folds of change. CAFs and NFs were isolated from oral cancer tissues and their normal counterparts. Cells (10^5) were suspended in PBS and incubated with different types of biotinylated lectins including L-PHA (2 mg/ml) and LEL (1 mg/ml) for 30 min followed by DyLight® 488 streptavidin staining for 30 min. For the Gal-1 binding analysis, recombinant Gal-1 was labeled with a DyLight labeling kit (Thermo Scientific) following the manufacturer's instructions, and 10^5 cells were incubated with fluorescence-labeled Gal-1 (Gal-1-488) for 30 min. The binding of different lectins to CAFs and NFs were analyzed with a BD Accuri™ C6 Cytometer (BD Biosciences). **(C)** NFs and CAFs were treated with different dosages of Gal-1 and the migration ability was measured by the Boyden chamber assay. *, $p<0.05$

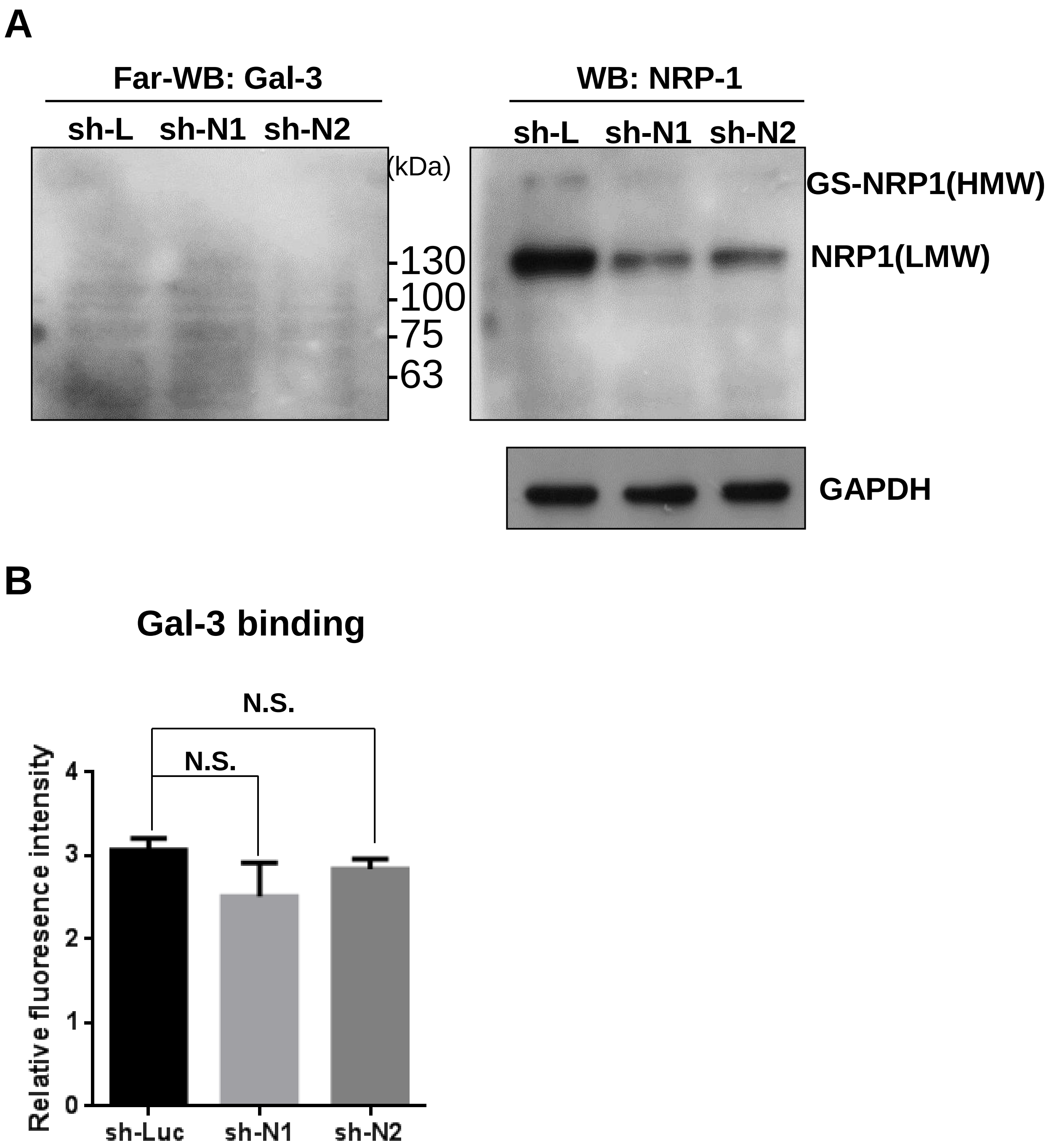

Figure S5. Galectin-3 (Gal-3) does not interact with neuropilin-1 (NRP-1). **(A)** No binding was observed between Gal-3 and NRP-1. The interaction between Gal-3 and NRP-1 was measured using Far-Western blotting as described in "Materials and methods". LX-2 cells were transduced with luciferase (sh-L) and neuropilin-1 shRNAs (sh-N1 and N2). The cell lysates were collected for Far-western blotting. **(B)** Recombinant Gal-3 was labeled with a DyLight labeling kit (Thermo Scientific) following the manufacturer's instructions, and 10^5 LX-2 cells were incubated with fluorescence-labeled Gal-3 for 30 min. The binding were analyzed with a BD Accuri™ C6 Cytometer (BD Biosciences).

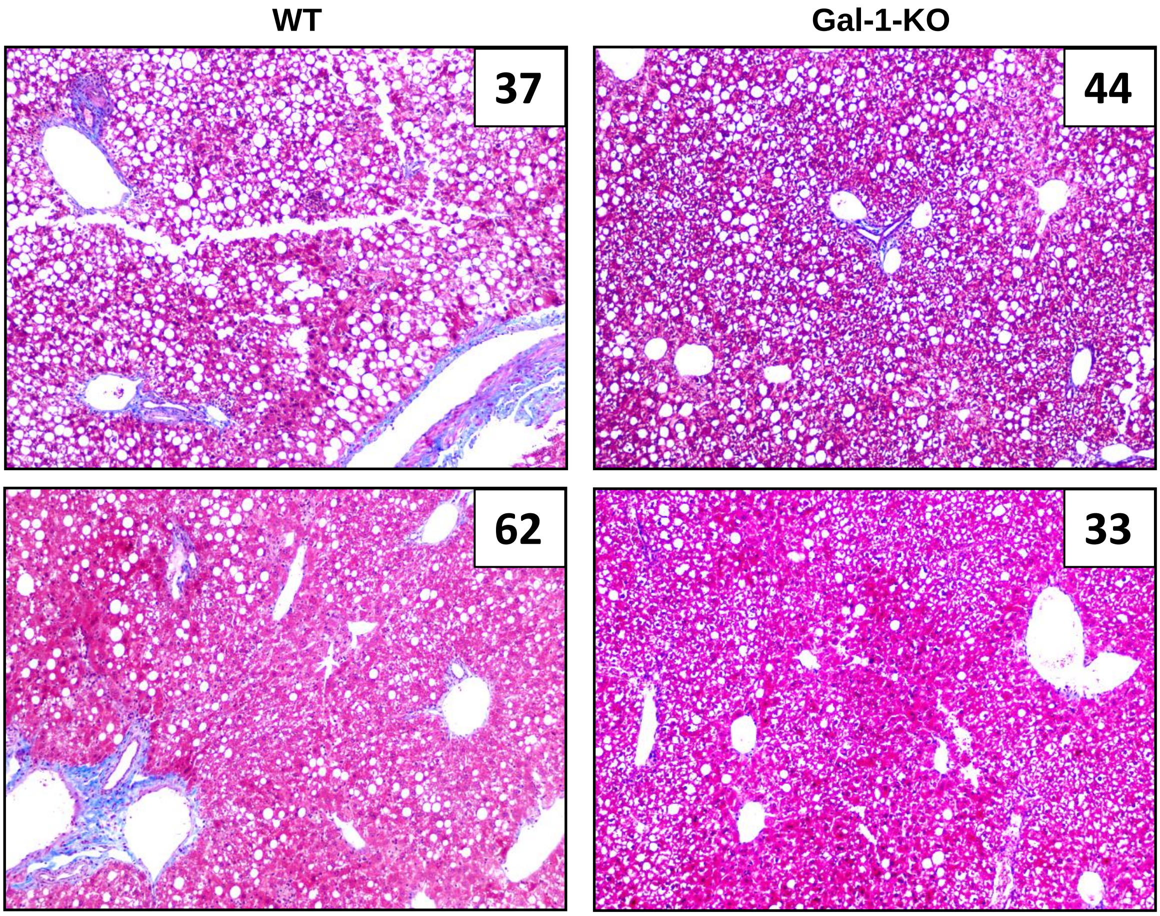

Figure S6. Masson's trichrome staining of wild-type (WT) and Gal-1 null mice (Gal-1-KO). Masson's trichrome staining was performed as the manufacture's instruction (Abcam). The blue color indicates collagen. No. 37 and 62 are WT mice. No. 33 and 44 are Gal-1-KO mice.

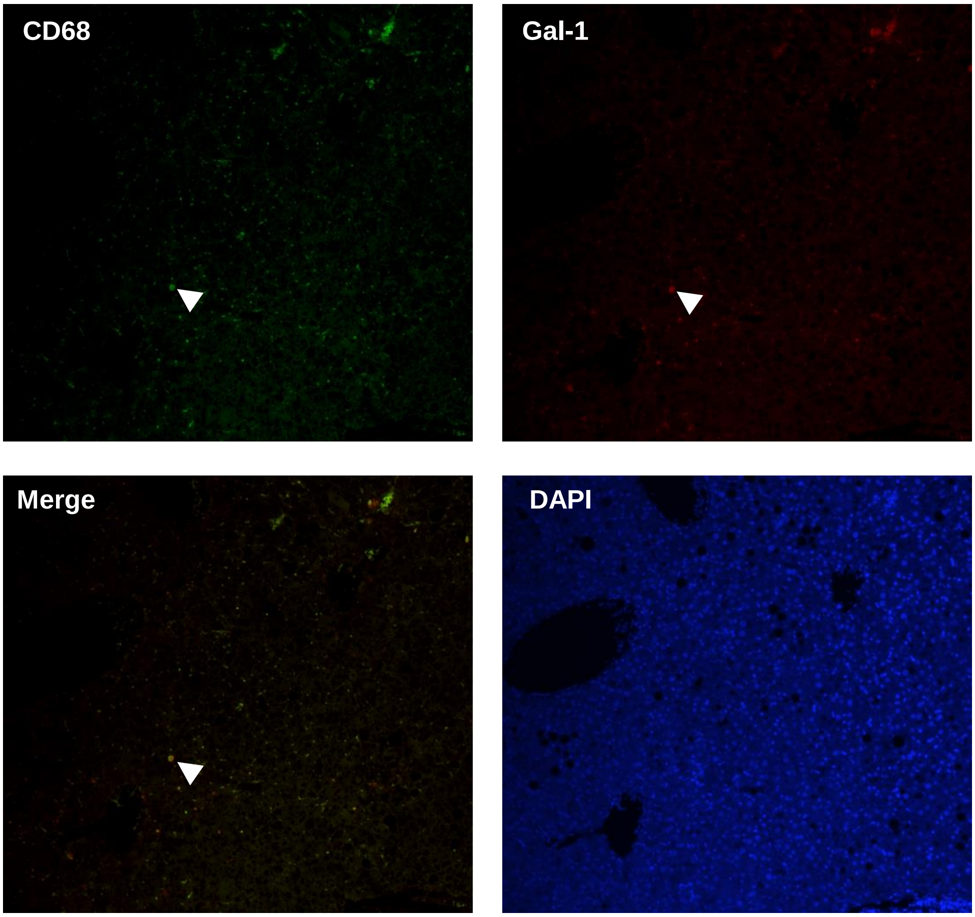

Figure S7. Immunofluorescence (IF) staining of MCD-fed mice. IF staining was performed as described in “Materials and methods”. The CD68 staining was co-localized with Gal-1 indicating macrophages expressed Gal-1. However, there were few CD68-positive signals in the livers indicating that the MCD feeding did not induce strong inflammatory responses in this model.

Figure S8. Full-length blots of figure 1A

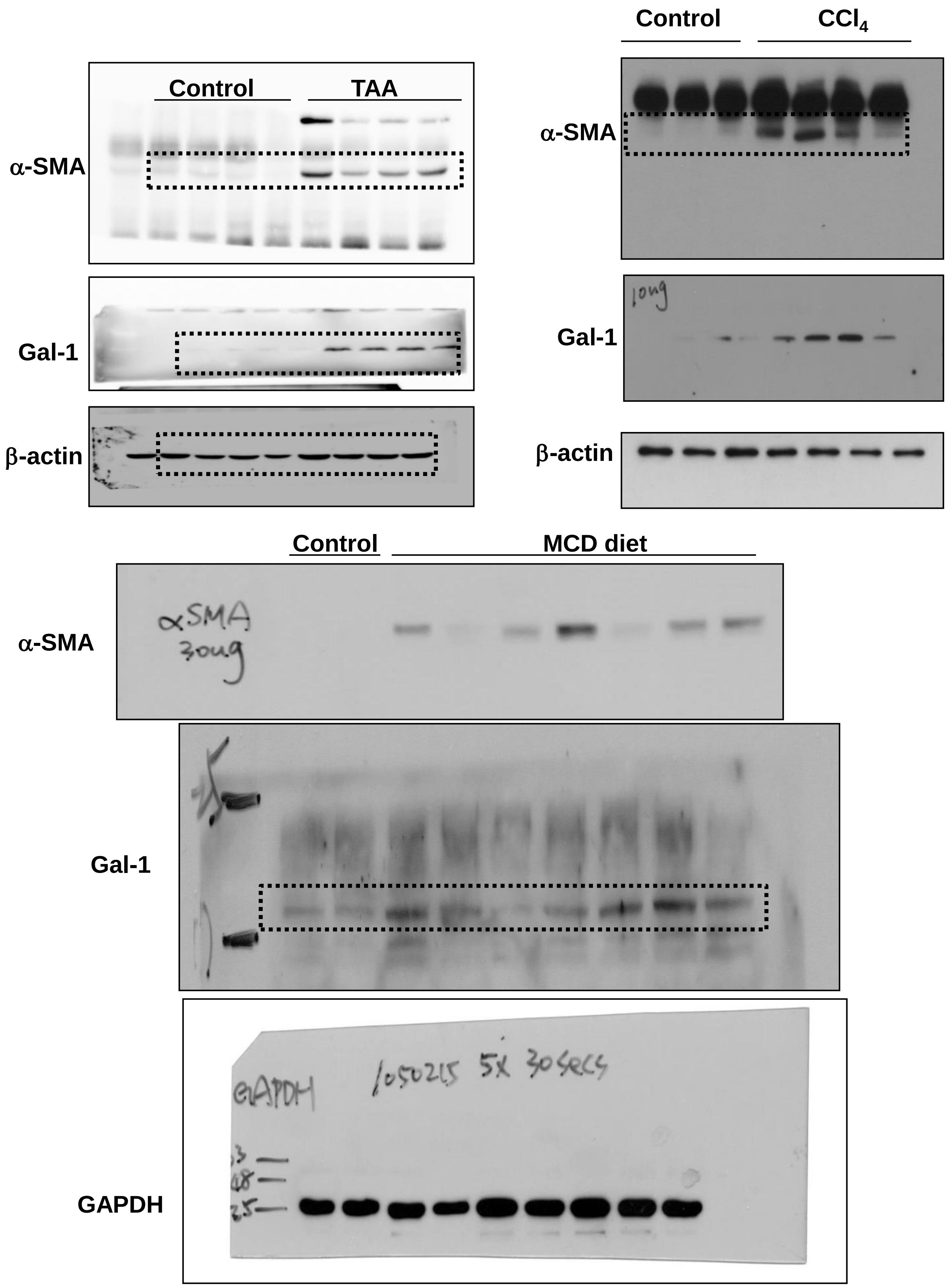

Figure S9. Full-length blots of figure 2E

α-SMA

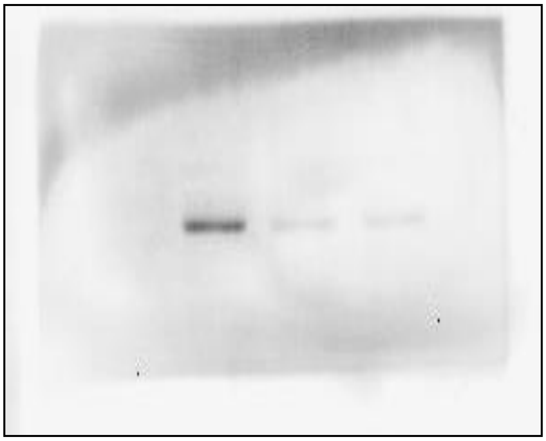

β-actin

Gal-1

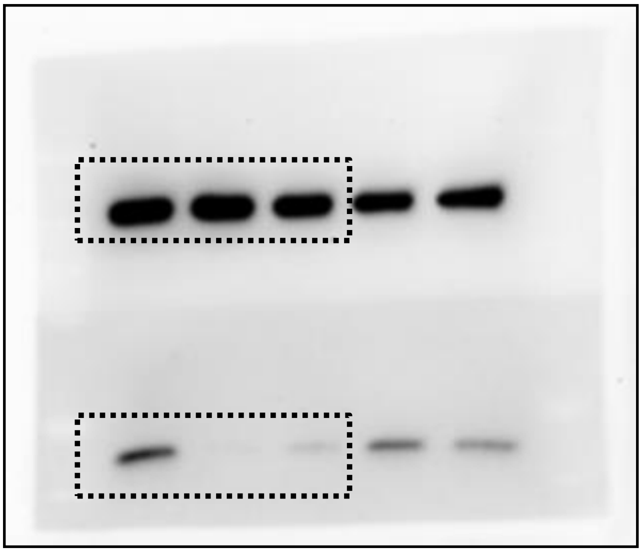

Fibronectin

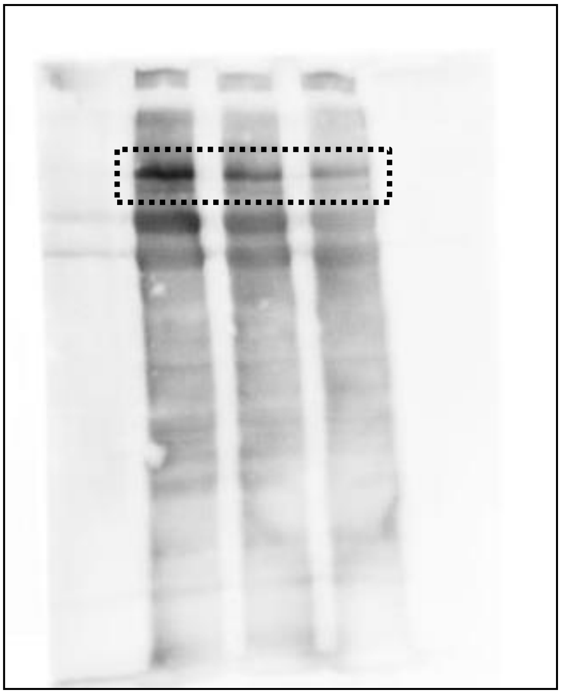

Figure S10. Full-length blots of figure 5A, D, E

Figure 5A

NRP-1

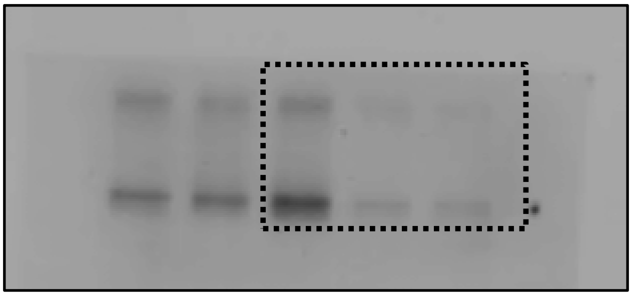

Figure 5E

Far-WB:
Gal-1

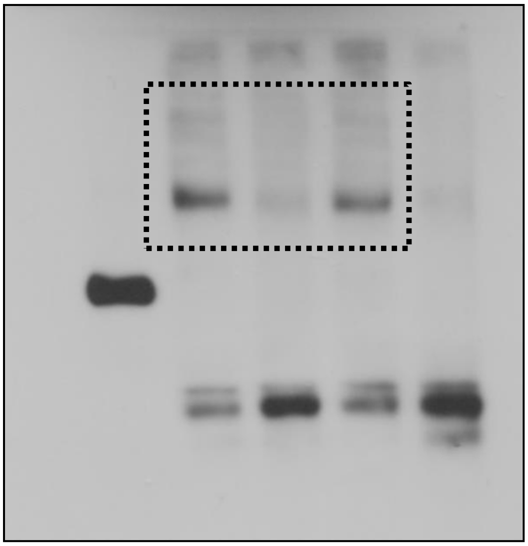

Figure 5D

Far-WB:
Gal-1

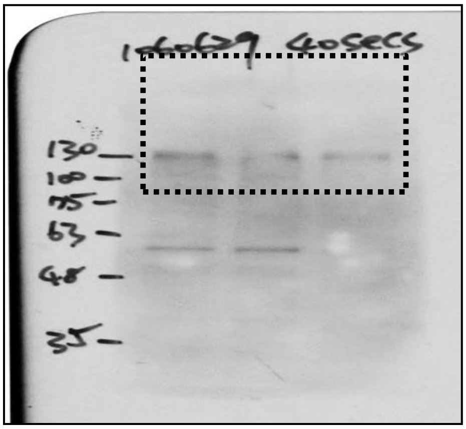

IB: NRP-1

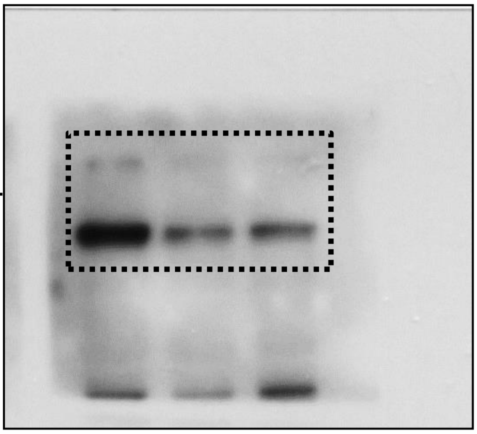

GAPDH

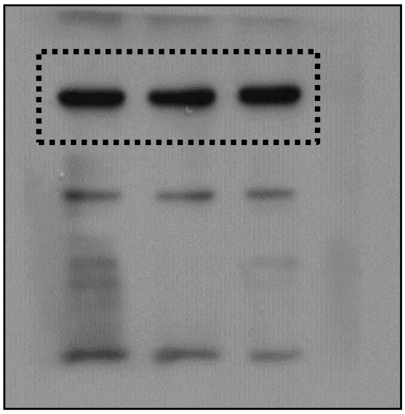

Figure S11. Full-length blots of figure 6A.

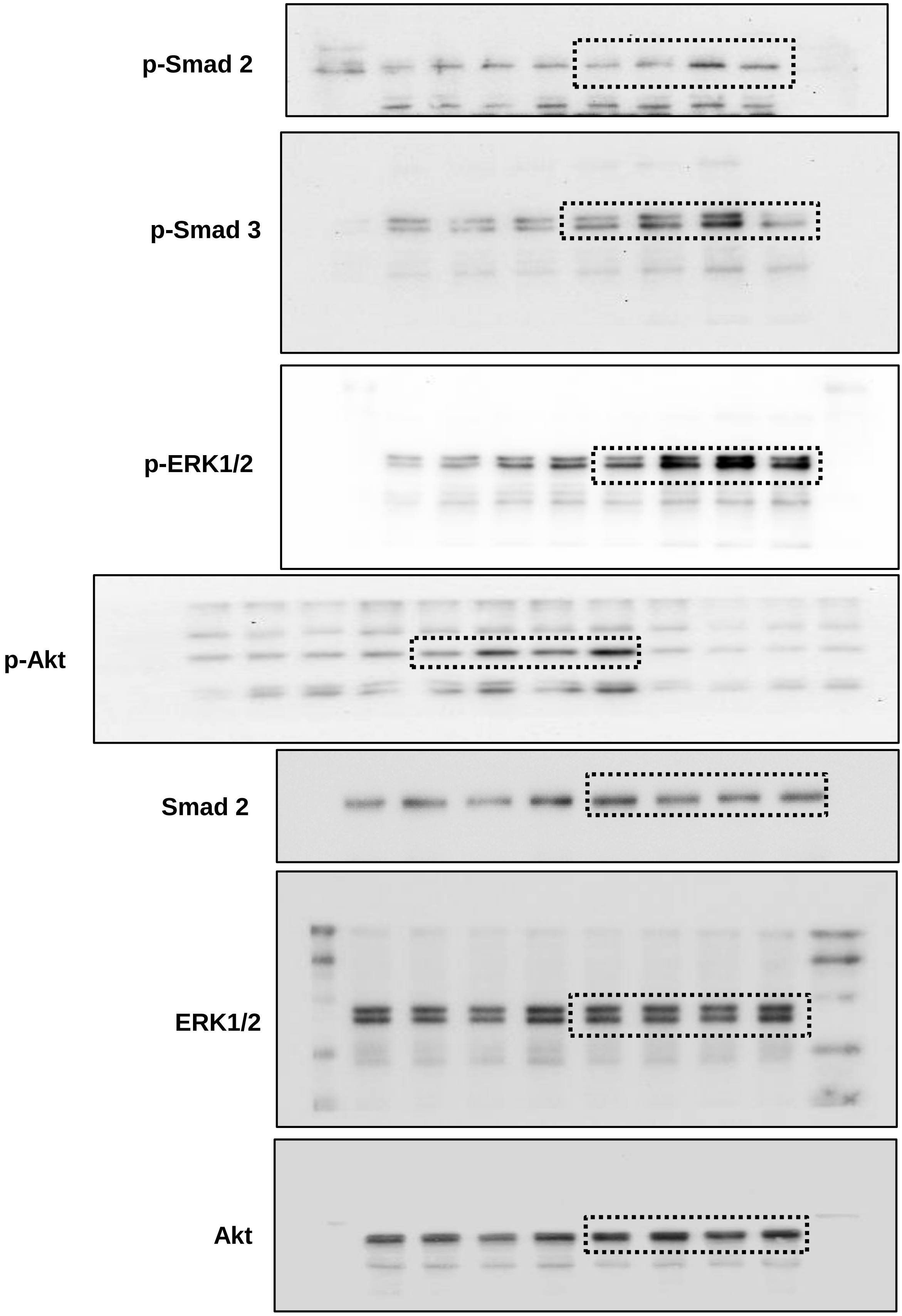

Figure S12. Full-length blots of figure 6B

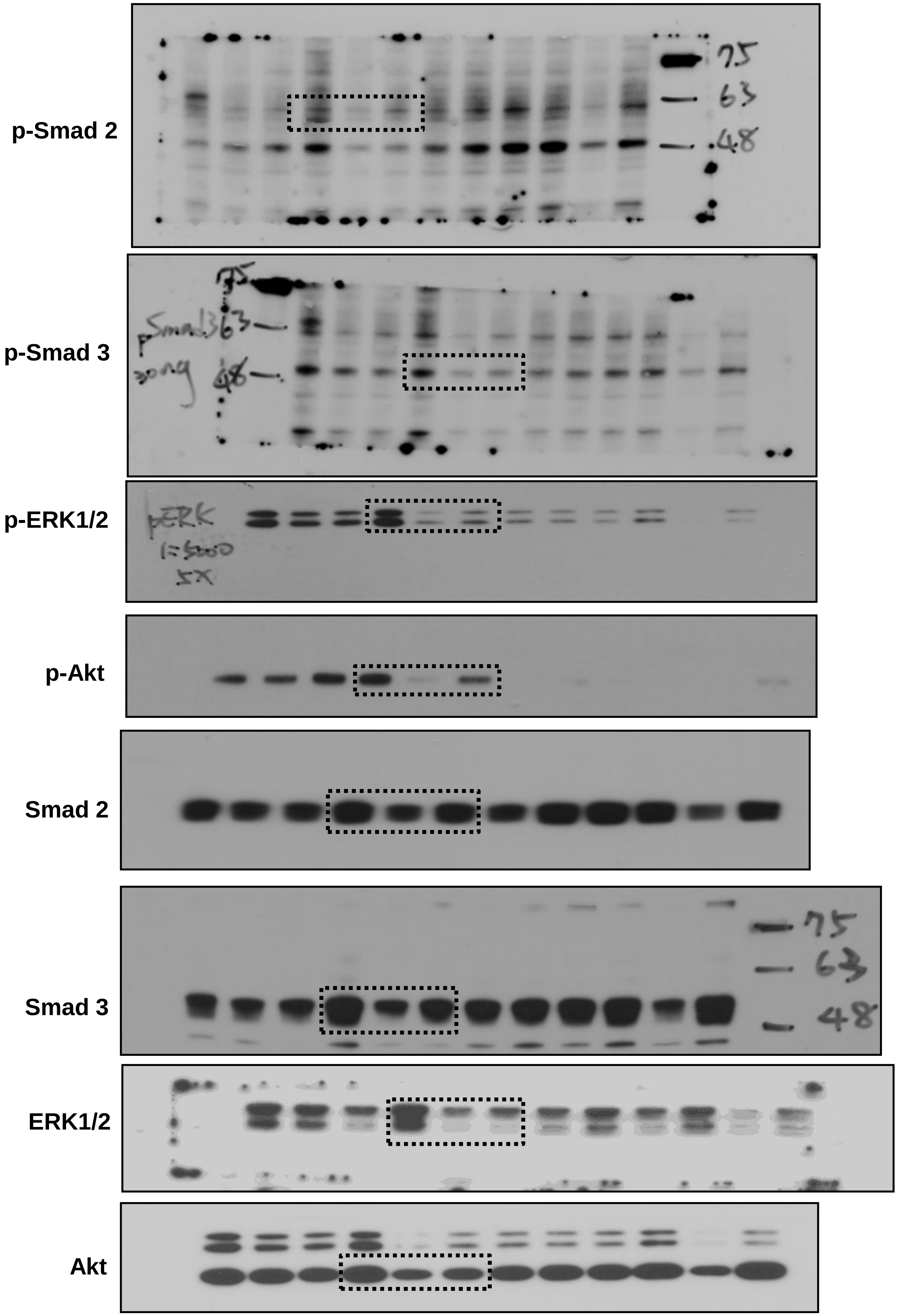

Figure S13. Full-length blots of figure 7A.

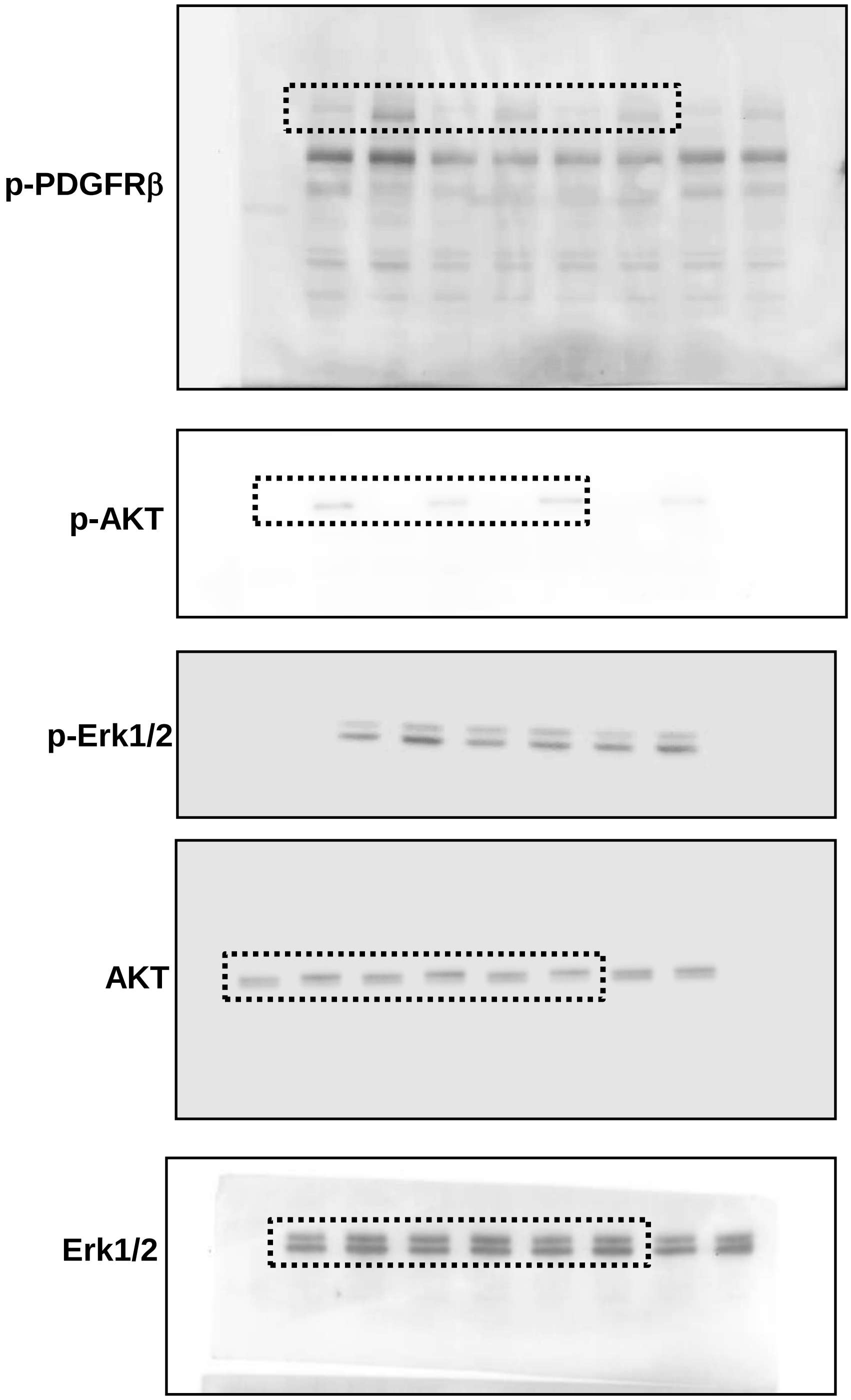

Figure S14. Full-length blots of figure 8A.

α-SMA

GAPDH

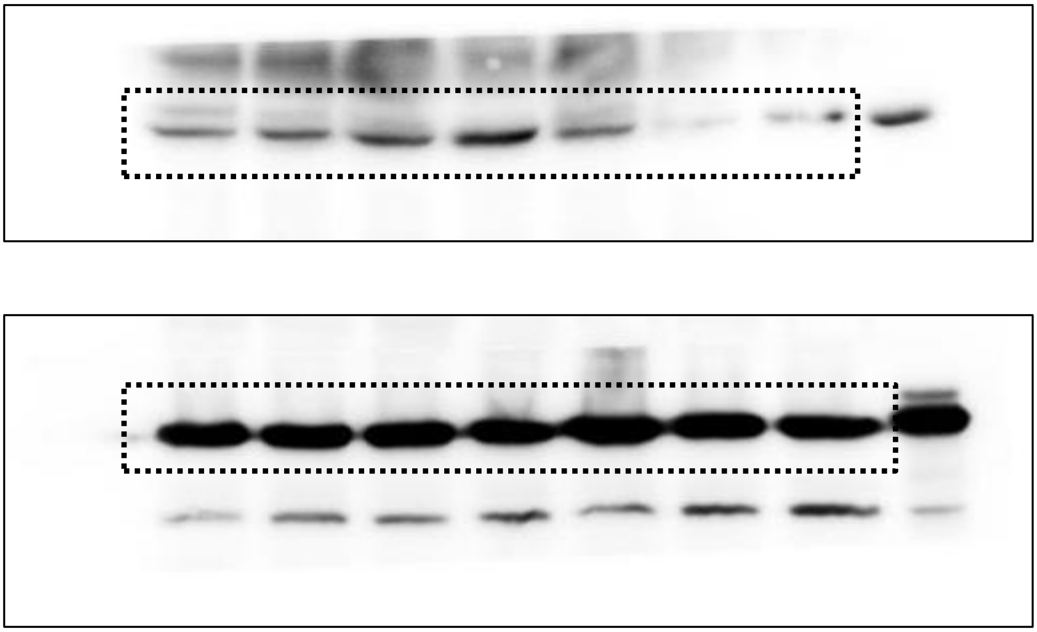

Supplement: Supplementary file 1 — Supplemental information [file 41598_2017_11212_MOESM1_ESM.pdf]
